# Spontaneous transitions between amoeboid and keratocyte-like modes of migration

**DOI:** 10.3389/fcell.2022.898351

**Published:** 2022-09-30

**Authors:** Ted Moldenhawer, Eduardo Moreno, Daniel Schindler, Sven Flemming, Matthias Holschneider, Wilhelm Huisinga, Sergio Alonso, Carsten Beta

**Affiliations:** ^1^ Institute of Physics and Astronomy, University of Potsdam, Potsdam, Germany; ^2^ Department of Physics, Universitat Politècnica de Catalunya, Barcelona, Spain; ^3^ Institute of Mathematics, University of Potsdam, Potsdam, Germany

**Keywords:** cell migration, amoeboid motility, keratocytle-like motility, modes of migration, *D. discoideum*, actin dynamics

## Abstract

The motility of adherent eukaryotic cells is driven by the dynamics of the actin cytoskeleton. Despite the common force-generating actin machinery, different cell types often show diverse modes of locomotion that differ in their shape dynamics, speed, and persistence of motion. Recently, experiments in *Dictyostelium discoideum* have revealed that different motility modes can be induced in this model organism, depending on genetic modifications, developmental conditions, and synthetic changes of intracellular signaling. Here, we report experimental evidence that in a mutated *D. discoideum* cell line with increased Ras activity, switches between two distinct migratory modes, the amoeboid and fan-shaped type of locomotion, can even spontaneously occur within the same cell. We observed and characterized repeated and reversible switchings between the two modes of locomotion, suggesting that they are distinct behavioral traits that coexist within the same cell. We adapted an established phenomenological motility model that combines a reaction-diffusion system for the intracellular dynamics with a dynamic phase field to account for our experimental findings.

## 1 Introduction

Actin-driven motility is an essential prerequisite for a wide range of biological functions, such as wound healing, immune responses, or embryonic development. The underlying signaling pathways and cytoskeletal structures may vary widely, depending on the cell type and biological context. Consequently, the associated cell morphodynamics and speeds of propagation are diverse, resulting in a wide range of different modes of motility. For example, the persistent locomotion of a fish keratocyte is clearly distinct from the more erratic motion of neutrophils or dendritic cells. In most cases, different modes of motility are linked to specific cell types and are closely related to their biological functions. However, there are also cases where migratory plasticity of the same cell type is an essential requirement for cellular function (Friedl and Wolf, 2003; Yamada and Sixt, 2019; Nikolaou and Machesky, 2020). For example, in order to achieve metastatic dissemination, cancer cells show a wide range of different migration strategies, including single celled amoeboid and mesenchymal motion as well as multicellular streaming and collective cell migration (Chambers et al., 2002; Psaila and Lyden, 2009; Friedl and Alexander, 2011). Here, individual cells undergo dramatic transformations to change their adhesion properties, remodel their actin cytoskeleton, and alter the activity of their associated signaling pathways (Vignjevic and Montagnac, 2008; Yilmaz and Christofori, 2010; Bear and Haugh, 2014). Despite these insights, many fundamental questions regarding migratory plasticity remain unresolved and call for experiments that rely on simpler model systems. In particular, a better understanding of switches in migratory modes will not only provide functional insights into processes such as cancer metastasis but will also elucidate whether specific dynamical actin structures are characteristics of individual cell types or universally emerge in different cellular contexts.

**FIGURE 1:**
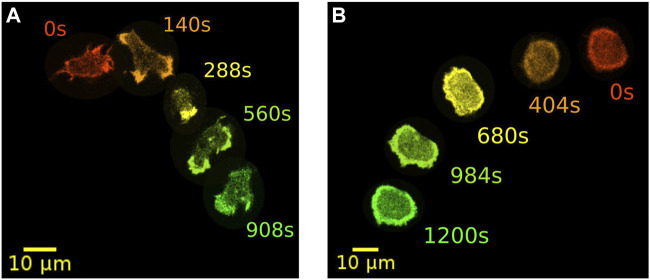
Examples of two distinct modes of motility. Amoeboid **(A)** and fan-shaped motion **(B)** of *D. discoideum* cells expressing Lifeact-GFP as a marker for filamentous actin. In each frame, five time points of the same cell track are displayed as an overlay. Time is color coded from red, orange and yellow to green.

A prominent model organisms to study actin-driven motility is the social amoeba *Dictyostelium discoideum* (*D. discoideum*). With its genome completely sequenced, *D. discoideum* is readily accessible for a wide range of biochemical and physiological studies. In addition, many parts of the receptor mediated signaling pathway and the downstream cytoskeletal machinery are conserved between *D. discoideum* and mammalian cells (Artemenko et al., 2014; Devreotes et al., 2017). It is well known that *D. discoideum* cells can exhibit other types of locomotion besides their common pseudopod-based amoeboid motility. In particular, they can move in a highly persistent fashion reminiscent of keratocytes. Here, pseudopodia are largely absent and cells display a stable, kidney-shaped morphology that is elongated perpendicular to their direction of motion. This so-called fan-shaped motility was first observed in *D. discoideum* cells lacking the *amiB* gene, which is required for aggregation in the course of starvation-induced development (Asano et al., 2004). In axenic wild-type cells, also the development under low cell density condition may increase the probability of observing fan-shaped cells (Cao et al., 2019b). Similarly, in a non-axenic DdB background, an increased proportion of fan-shaped cells can be induced by deletion of the *axeB* gene that encodes the RasGAP NF1 (Flemming et al., 2020; Moreno et al., 2020). Switches from amoeboid to fan-shaped motility could be even directly induced by decreasing phosphatidylinositol-4,5-bisphosphate (PtdIns(4,5)P_2_) levels or by increasing Ras/Rap activity with the help of a rapamycin-induced dimerization system (Miao et al., 2017). In Figure 1, examples of NF1-deficient DdB cells are displayed, exhibiting amoeboid (left) and fan-shaped motility (right) for comparison.

In fan-shaped *D. discoideum* cells, a large fraction of the substrate-attached ventral membrane is filled with a composite PtdIns(3,4,5)P_3_ (PIP_3_)/actin wave that drives the persistent forward motion (Asano et al., 2008). This was confirmed by the observation of wave-driven cytofission events in oversized *D. discoideum* cells, where it can be clearly seen how the driving wave segment covers the bottom cortex of the emerging daughter cell that moves in a fan-shaped fashion (Flemming et al., 2020). The fan-shaped mode is thus intrinsically linked to the presence of basal actin waves. Consequently, treatment with an inhibitor of PI3-kinase (LY294002) that suppresses the formation of basal waves results in a breakdown of fan-shaped motility (Asano et al., 2008; Miao et al., 2017; Flemming et al., 2020). The emergence of actin waves in *D. discoideum* is associated with increased macropinocytic activity, which arises in cells showing hyper Ras activity as a consequence of an NF1 deficiency (Veltman et al., 2016). This is in line with the observation that fan-shaped cells can be induced by synthetically increasing Ras/Rap activity (Miao et al., 2017).

In previous experiments, where fan-shaped motility was induced by changing developmental conditions or by introducing knockouts, typically only a fraction of the entire cell population adopted the fan-shaped mode (Asano et al., 2004; Cao et al., 2019b). This can be attributed to heterogeneity within the cell population, such as, for example, cell-to-cell variability in gene expression levels, developmental state, or different distributions of other intracellular parameters. Here we report that switches between amoeboid and fan-shaped modes of migration may also occur spontaneously within the same cell, and we provide a detailed analysis of this reversible switching process. Specifically, when NF1 is knocked out in the non-axenic DdB background, fan-shaped cells are only transiently stable, and cells switch back and forth between fan-shaped and amoeboid modes. The transition from amoeboid to fan-shaped motility requires the nucleation of an actin wave that grows and eventually fills most of the ventral cortex of the cell to form a stable fan that lives until spontaneous breakdown of the wave occurs. Not every growing wave successfully locks into a stable fan-shaped configuration, indicating that both the amoeboid and the fan-shaped modes are distinct coexisting states of cortical actin organization. To account for these observations, we propose an extension of a well-established stochastic reaction-diffusion model of cortical wave dynamics that incorporates bistability of amoeboid and fan-shaped states.

## 2 Materials and methods

### 2.1 Cell culture

For all experiments the non-axenic *D. discoideum* strain DdB NF1 KO was used (Bloomfield et al., 2015) that carries a knockout of the *axeB* gene encoding a homologue of the human RasGAP NF1. The deficiency in NF1 leads to a phenotype that results in a high percentage of fan-shaped cells (Flemming et al., 2020). Cells were cultivated in 10 cm dishes or in 125 ml Erlenmeyer flasks with Sørensen’s buffer (14.7 mM KH_2_PO_4_, 2 mM Na_2_HPO_4_, pH 6.0), supplemented with 50 *μ*M MgCl_2_, 50 *μ*M CaCl_2_, and G418 (5 *μ*g/ml) as selection marker for the NF1 KO. The suspension also contained *Klebsiella aerogenes* at a final OD_600_ of 2, which was achieved by adding a concentrated *Klebsiella aerogenes* solution with an OD_600_ of 20 in a volume corresponding to 1/10 of the final volume of the suspension. The bacteria were grown in shaking LB medium in a volume of 1 L to a final OD_600_ of 2, washed 3 times in Sørensen’s buffer, and then resuspended in Sørensen’s buffer to a concentrated solution with an OD_600_ of 20.

The DdB NF1 KO cells were transformed with an episomal plasmid encoding for Lifeact-GFP (either SF99 or SF108) as F-actin marker (Flemming et al., 2020). The expression vectors are based on a set of vectors for gene expression in non-axenic cells (Paschke et al., 2018) and were described in Flemming et al. (2020). The plamids were transformed into DdB NF1 KO cells by electroporation as described before with an ECM2001 electroporator using three square wave pulses of 500 V for 30 ms in electroporation cuvettes with a gap of 1 mm. Hygromycin (33 *μ*g/ml) was used as selection marker for the expression plasmid (Flemming et al., 2020).

### 2.2 Image acquisition

In experiments with the DdB NF1 KO strain, an increased fraction of fan-shaped cells can be observed during the aggregation stage of the *D. discoideum* lifecycle (Moreno et al., 2020). Aggregation was initiated by removing *Klebsiella aerogenes* to initiate starvation of the *D. discoideum* cells. DdB NF1 KO cells were grown over night in shaking culture in a volume of 25 ml starting from a density of 3 × 10^6^ cells/ml to a final concentration of 3 × 10^7^ cells/ml. The next day, remaining bacteria were removed by washing the cells 3 times with Sørensen’s buffer (centrifugation at 300 × g). After washing, the cells were resuspended in 25 ml of Sørensen’s buffer and incubated for 3–6 h at 200 rpm in a shaking incubator at 22°C. During this incubation time, bacteria that remained after washing were taken up by the *D. discoideum* cells and the development was initiated. Cells were harvested by centrifugation (300 × g) and transferred into a 35 mm microscopy dish with glass bottom (FluoroDish, World Precision Instruments) for imaging. The cells were diluted to a density that enabled the imaging of single cell tracks. A Laser Scanning Microscope (LSM 780, Zeiss, Jena) with a 488 nm argon laser and either a 63 × or 40 × oil objective was used for imaging. We observed 48 events, where cells spontaneously switched between amoeboid and fan-shaped modes in a total number of 94 recordings that were each taken over 38 min on average.

### 2.3 Data analysis

First, discrete sets of points approximating the cell contours were extracted from microscopy images using a modified version of the active contour (snake) algorithm described in (Driscoll et al., 2011; Driscoll et al., 2012; Lepro, 2021). An implementation of this segmentation approach can be found in AmoePy, a Python-based toolbox for analyzing and simulating amoeboid cell motility (Schindler et al., 2021a). We then used AmoePy to compute smooth representations of the cell contours and the corresponding kymographs of the contour dynamics, see Supplementary Figure S1 for an explanation of the kymograph concept. The mathematical framework underlying AmoePy was introduced in (Schindler et al., 2021b). In short, a Gaussian process regression was used to obtain smooth estimates of the cell contour. Then, reference points on these contours (so-called virtual markers) were tracked in time relying on a one-parameter family of regularizing flows. Expansions and contractions were identified based on a novel quantity, the local dispersion. It measures the local stretching rate of virtual markers on the contour, see Supplementary Figure S1 for an illustration. For this reason, the local dispersion displays a clearly distinct behavior for the two different types of motility: A strong local thinning of markers under the narrow and elongated amoeboid protrusions; and an almost even distribution of markers at the broad leading edge of fan-shaped cells. These characteristics can be observed, for example, in Figure 4C, where the local dispersion kymograph displays large values during amoeboid migration (until *t* = 1,000 s), reflected by dark red and dark blue patches. After switching to the fan-shaped mode, the local dispersion takes small values close to zero, indicated by green patches, confirming that virtual marker thinning is less prominent in this state.

To determine the actin wave area *A*_*W*_, we applied an intensity threshold to the fluorescence images, using the “Threshold” function of ImageJ.“Threshold values were chosen based on visual inspection for each image sequence individually. The wave area was then determined by summing up the pixels exceeding the threshold value. The fluorescence intensity kymographs in Figure 3D; Supplementary Figures S4D,S5D were produced with the ImageJ “Multi Kymograph” function.

### 2.4 Numerical simulations

The equations of our mathematical model are presented in the Supplementary Appendix (Presentation 2). Eqs 1–7 were numerically integrated with periodic boundary conditions using central finite differences. The pixel size was defined as Δ*x* = 0.15 *μ*m with an integration step of Δ*t* = 0.002 s. For the temporal integration of the stochastic partial differential equations we have employed the Euler-Maruyama method. The total area considered for each cell was initialized at *A*_0_ = 113 *μ*m^2^ corresponding to a circular cell with radius *r* = 6 *μ*m. To produce polarized cells that are immediately motile, initial conditions were chosen such that the activatory component *c* is asymmetrically distributed across a part of the cell area, see Supplementary Figure S2. The parameters as well as the values and definitions can be found in Supplementary Table S1.

## 3 Results

### 3.1 Cells spontaneously switch between stable amoeboid and fan-shaped modes of migration

**FIGURE 2:**
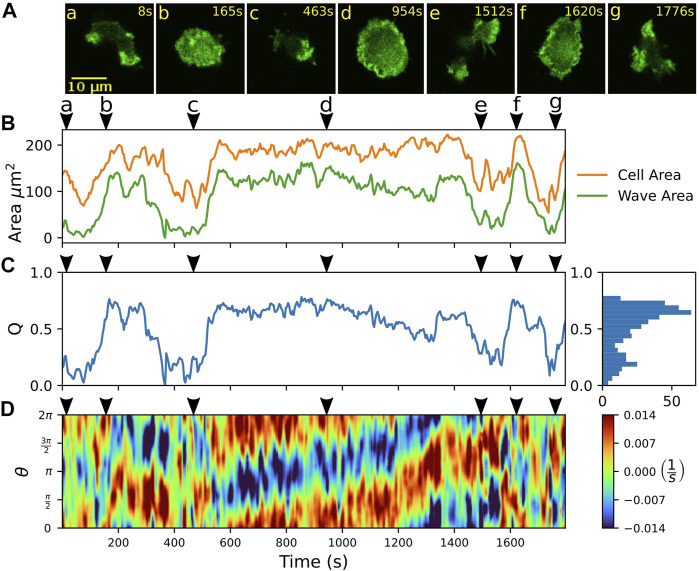
Spontaneous switching between motility modes. **(A)** Fluorescence images of a Lifeact-GFP expressing cell, alternating between amoeboid **(a,c,e,g)** and fan-shaped mode **(b,d,f)**. **(B)** Time trace of projected cell area *A*_*C*_ (orange) and actin wave area *A*_*W*_ (green). **(C)** Time trace of the relative wave area *Q* = *A*_*W*_/*A*_*C*_, where high values are associated with fan-shaped and low values with amoeboid motion. The histogram on the right shows the frequency of *Q*-values. **(D)** Kymograph of the local dispersion along the cell contour. Red (blue) indicates extending (contracting) regions along the contour, associated with protruding (retracting) parts. Arrowheads above the kymographs mark the time points corresponding to the fluorescence images in **(A)**.

Previously, stable fan-shaped cells have been observed as a consequence of genetic modifications, specific developmental conditions, or synthetic changes in the localization of intracellular signaling components (Asano et al., 2008; Miao et al., 2017; Cao et al., 2019b). Here, we report that *D. discoideum* cells may also show spontaneous, reversible switches between amoeboid and fan-shaped motility. Our observations were recorded in non-axenic DdB cells, where the *axeB* gene was disrupted, so that the RasGAP NF1 could not be expressed and, consequently, Ras activity was increased (Veltman et al., 2016). The spontaneous switches occurred in the developed state after two to 6 h of starvation. In Figure 2, an example is displayed. The sequence of fluorescence images in Figure 2A shows a cell undergoing a series of several consecutive switching events.

Besides manual classification based on visual inspection by an observer, switches between amoeboid and fan-shaped modes can be identified by changes in the projected cell area *A*_C_ (Miao et al., 2017). In Figure 2B, the area *A*_C_ is shown over time (orange line). In the fan-shaped state, the cell adopts a flattened, spread-out geometry. This leads to high values of *A*_C_ for the fan-shaped state, in contrast to lower values, resulting from the more compact geometry during amoeboid migration. As the fan-shaped state is associated with an actin wave that spreads across the ventral cortex, we also determined the cortical area *A*_W_ that is covered by the actin wave at each time point of our recordings, see the green line in Figure 2B. This was accomplished by expressing a fluorescent marker for filamentous actin (Lifeact-GFP), thus waves could be identified based on increased intensity values in the fluorescence images. Qualitatively, the temporal evolution of the wave area *A*_W_ follows the same trend as the projected cell area *A*_C_. As the absolute values of cell and wave areas may differ from cell to cell, we introduced the dimensionless ratio *Q* = *A*_W_/*A*_C_, which we call the relative wave area, to distinguish the two migration modes. In Figure 2C, the time evolution of the relative wave area *Q* is shown. The *Q*-values are confined between 0 and 1 because the wave area *A*_W_ cannot exceed the projected cell area *A*_C_. Typically, values of *Q* < 0.5 are associated with the amoeboid mode of motion, whereas values of *Q* > 0.5 indicate a fan-shaped state. The two states are reflected by two peaks in the frequency histogram of *Q*-values that is displayed on the right-hand side of Figure 2C. This classification into amoeboid and fan-shaped cells based on the relative wave area *Q* is not specific to our data set but can be generally applied also when these migratory modes are induced by other conditions. To demonstrate this, we have calculated *Q*-values from previously published data by others, where amoeboid and fan-shaped modes were induced separately by synthetic clamping of the intracellular PtdIns(4,5)P_2_ levels (Miao et al., 2017; Ghabache et al., 2021). The Q-values of amoeboid and fan-shaped cells from these data sets are in good agreement with the *Q*-values that were observed in our own experiments, see Supplementary Figure S3.

The kymograph in Figure 2D displays the local dispersion computed for 400 virtual markers placed along the cell circumference that was parameterized by an angular coordinate ranging from 0 to 2*π*. The dispersion is a measure of the local stretching rate of the contour and provides a sensitive measure to identify protruding and retracting cell border regions (Schindler et al., 2021b). In particular, positive (negative) values of the local dispersion correspond the protruding (retracting) parts of the contour. For cells that move in a fan-shaped fashion, the leading and trailing edges are reflected by broad regions in the kymograph that show persistently protruding and retracting activity, respectively, see for example the fan-shaped episode between 600 and 1,300 s in Figure 2D. In contrast, amoeboid movement results in a random distribution of smaller patches of activity along the contour that can be associated with extending and retracting pseudopodia. Note that also at the leading edge of fan-shaped cells, occasionally small short-lived protrusions emerge. However, they typically do not interfere with the overall stable movement of the fan-shaped cell, see Supplementary Figure S4 for an example.

### 3.2 Fan-shaped cells are formed by wave nucleation and growth from amoeboid cells

**FIGURE 3:**
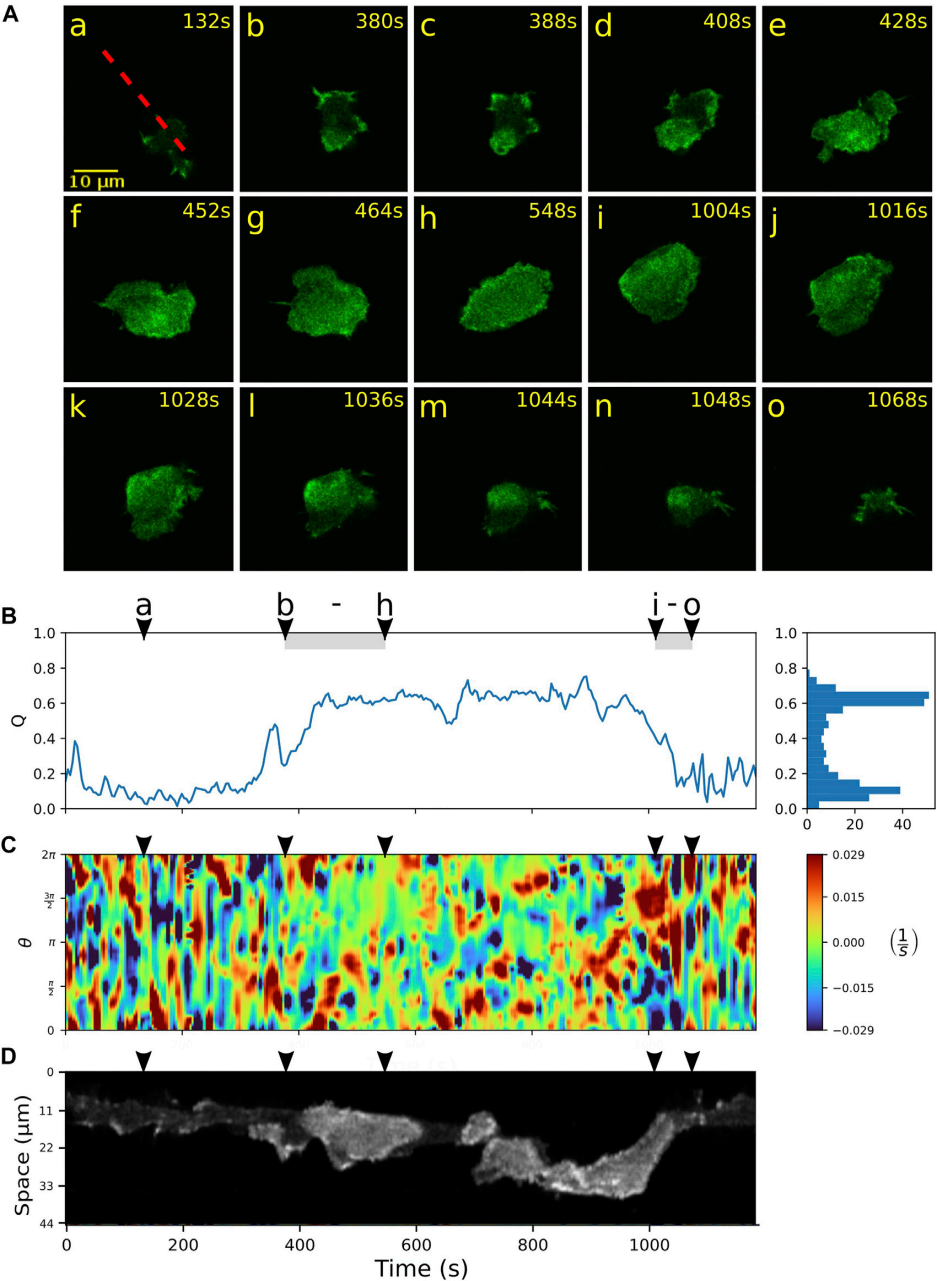
Growth and decay of actin waves mediate transitions between amoeboid and fan-shaped modes. **(A)** Starting from a cell in amoeboid mode **(a)**, nucleation and growth of an actin wave is shown in **(b–h)**, followed by a rapid wave breakdown in **(i–o)**. **(B)** Time evolution of the relative wave area *Q*. The shaded intervals belong to the episodes of actin wave growth **(b–h)** and decay **(i–o)**. Histogram on the right-hand side shows the frequency of *Q*-values. **(C)** Kymograph of the local dispersion along the cell contour. **(D)** Fluorescence kymograph taken along the red dashed line shown in the first panel of **(A)**. Arrowheads above the kymographs indicate the time points of the corresponding fluorescence images in **(A)**.

We further focus on a more detailed characterization of the switching events between amoeboid and fan-shaped modes of locomotion. Typically, the bottom cortex of DdB wild type cells exhibits a dynamically fluctuating F-actin density with long-lived actin foci and occasional bursts of larger actin patches that transiently emerge and decay (Flemming et al., 2020). In contrast to the wild type, in NF1 KO cells that show hyper Ras activity, these actin patches may increase in size and become the nucleus of a growing ring-shaped actin wave. In Figure 3A, an example of such an event is shown. Starting from the amoeboid state (a), a wave is nucleated close to the lower border of the cell (b), grows in size (c,d), and spreads across the ventral cortex until the entire projected area of the cell is filled by the actin wave (h). During this growth process, the actin wave pushes the cell border outwards, eliminating smaller pseudopodial protrusions and irregularities of the cell shape, until it converges to the roundish, spread-out morphology of a fan-shaped cell. This is reflected by an increase in the relative wave area *Q* from frame (b) to (h) in Figure 3B. During the same time interval, the kymograph of the local dispersion in C shows that the numerous irregular protrusions that can be observed during amoeboid migration, are increasingly suppressed, giving rise to a smoother cell contour with only a few broader protrusions. A fluorescence kymograph taken along the dashed red line in (a) confirms that this time interval coincides with a growing wave, as can be seen by the increased intensity levels of the Lifeact-GFP label inside the wave area, see Figure 3D.

Once a fan-shaped geometry has been reached, we observed two different scenarios for the further evolution. On the one hand, the extended wave can be unstable, giving rise to breakup of the wave area and an eventual decay back to the amoeboid state. During this process, the relative wave area *Q* fluctuates around an elevated level, see Figure 3B between (h) and (i), accompanied by an irregular dynamics of the cell contour displayed in C. The fluorescence kymograph in panel D confirms that the wave area is unstable during this time interval. The final return to the amoeboid state is associated with a complete decay of the wave between time frames (i) and (o) and can be identified by a steep decrease in the relative wave area *Q* in panel B.

**FIGURE 4:**
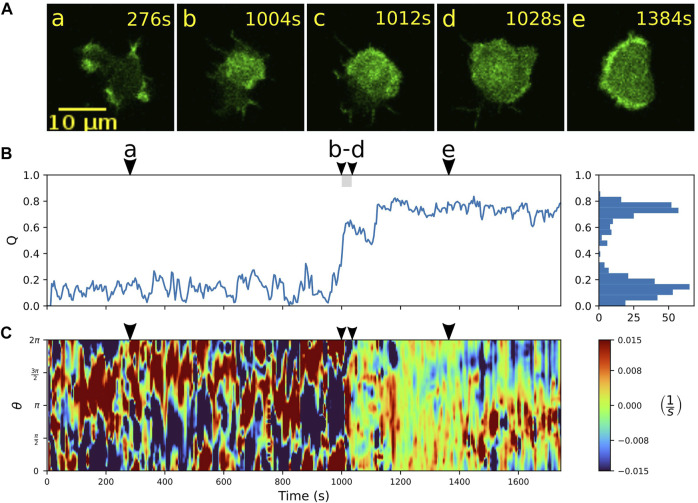
Transition from amoeboid to fan-shaped mode. **(A)** Sequence of fluorescence images starting in amoeboid mode **(a)** and switching **(b–d)** into the fan-shaped mode **(e)**. **(B)** Time evolution of the relative wave area *Q*. Histogram on the right-hand side shows the frequency of *Q*-values. **(C)** Local dispersion kymograph taken along the cell contour. Arrowheads above the kymographs indicate the time points of the corresponding fluorescence images in **(A)**.

Alternatively, once the wave fills the entire bottom cortex, the cell can “lock” into a stable fan-shaped state, characterized by a large, extended wave segment that maintains a roundish, elongated morphology and pushes the cell forward in a persistent fashion. Details of the transition to a stable fan can be seen in Figure 4. Starting from an amoeboid state (a), a rapid transition to the fan-shaped mode induced by a growing wave is captured in time frames (b) to (d), which is accompanied by an increase in the relative wave area *Q* and a smoothing of the cell contour, see Figures 4B,C. In this case, the fan initially remains stationary and resumes movement only after 1,400 s, as can be seen from the formation of a persistent leading edge in the local dispersion kymograph in panel C (red patches steadily located between 0 and *π* on the contour). The transition from fan-shaped back to amoeboid motility is typically initiated by a spontaneous breakdown of the basal actin wave, see Supplementary Figure S5 for an example. It proceeds in a similar manner as the collapse of an unstable fan shown in Figure 3.

### 3.3 Numerical simulations of a phenomenological motility model capture the experimental findings

We performed numerical simulations using a well-established phenomenological model of actin-driven cell motility (Alonso et al., 2018). The model is based on a noisy bistable reaction-diffusion system for the intracellular dynamics that is combined with a dynamic phase field to take the evolution of the cell shape into account, see the Supplementary Appendix (Presentation 2) for a presentation of the model equations. The patterns of cortical activity are modeled by a single dynamical species *c* that can be associated with activatory components of the signaling pathway, such as activated Ras, PI3K, and PIP_3_. Thus, the component *c* indirectly reflects also the cortical F-actin patterns that drive the cell contour dynamics, which is achieved in the model by a coupling of the local concentration of *c* to the dynamics of the confining phase field.

We previously found that this model shows both amoeboid and fan-shaped motility modes depending on the choice of model parameters, such as the noise intensity and the average cortical coverage with the activatory component *c* (Moreno et al., 2020). This and previous findings by others suggest that within a cell population, different motility modes may arise due to cell-to-cell variability (Asano et al., 2004; Cao et al., 2019b). However, considering our experimental observation that individual cells frequently switch back and forth between both modes of locomotion, we assume that these modes are coexisting behavioral traits within the same cell, so that noise induced transitions may occur between them.

In order to take these findings into account, we extended our previously established model. Specifically, the average cortical coverage with the activatory species *c*, *C*_0_ = *Q*_0_ *∫ϕ dA*, that was a parameter in the previous version of the model, can now dynamically change between two stable states of low and high *c*-coverage, respectively, see Eq. A6. The low coverage state corresponds to the amoeboid mode, where the cortical activity is dominated by small, short-lived bursts of actin activity, whereas the high coverage state is associated with the fan-shaped mode, where most of the ventral cortex is covered by a large, stable actin wave.

**FIGURE 5:**
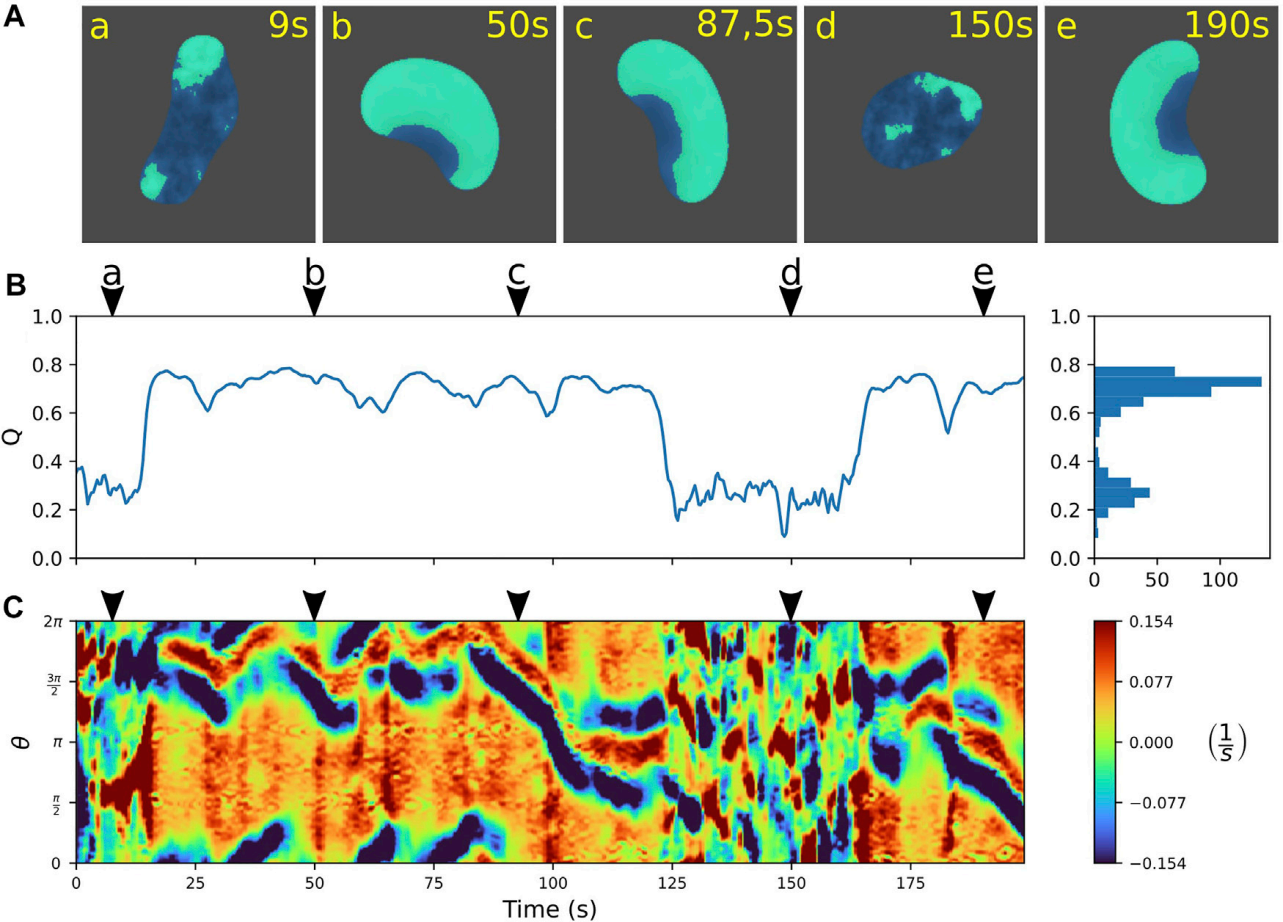
Predicting spontaneous switching between motility modes by the model. **(A)** Snapshots of a cell alternating between amoeboid **(a,d)** and fan-shaped mode **(b,c,e)**. **(B)** Time trace of the relative wave area *Q* = *A*_*W*_/*A*_*C*_, where high values are associated with fan-shaped and low values with amoeboid motion. Histogram on the right shows the frequency of *Q*-values. **(C)** Kymograph of the local dispersion taken along the cell contour. Arrowheads above the kymographs mark the time points corresponding to the snapshots in **(A)**. The concentration constraint parameter was set to *M* = 0.0015 and the threshold value to *Q*_*Th*_ = 0.45; for all other model parameters see Supplementary Table S1.

In numerical simulations of the extended model, we indeed observed rapid, spontaneous switches between amoeboid and fan-shaped locomotion. An example is displayed in Figure 5, where the individual snapshots shown in panel A can be assigned to either the amoeboid mode that shows small irregular patches of cortical activity (a and d), or to the fan-shaped mode that is dominated by a large wave segment, imposing a stable, elongated cell shape (b, c, and e). Similar to the experimental observations, also in simulations the two states can be distinguished based on the value of the relative wave area *Q*. In Figure 5B, the time evolution of *Q* is displayed, clearly highlighting the transitions between the two stable states. They are characterized by their respective stable *Q*-values. Note that, in the numerical simulations, the *Q*-values of the amoeboid and fan-shaped states correspond to the average cortical coverages *Q*_0_ in the respective states. They are model parameters chosen in agreement with the experimental observations, see Eq. A6.

To further characterize the dynamics of the simulated cell contours, we computed kymographs of the local dispersion. Similar to the experiments, the contour evolution is dominated by small irregular protrusions in the amoeboid state that is distinct from the regular pattern with stable leading and trailing edges in the fan-shaped state, see Figure 5C.

Additional numerical examples of switching events between amoeboid and fan-shaped locomotion can be seen in the Supplementary Material (Presentation 1), where Supplementary Figure S6 shows a switch from amoeboid to fan-shaped mode and Supplementary Figure S7 a transition from a fan to an amoeba, induced by spontaneous breakdown of the driving wave segment. To further asses the performance of our model, we analyzed the shape of amoeboid and fan-shaped cells by measuring the distance from each point on the cell border to the center of mass of the cell and compared the time evolution of this quantity for experimentally measured and numerically simulated cells. The resulting kymographs and profiles are displayed in Supplementary Figures S9, S10. They show that the shape evolution of both the rapidly changing, irregular amoeboid cells as well as the stable elongated fan-shaped cells is closely reproduced by our model simulations.

**FIGURE 6:**
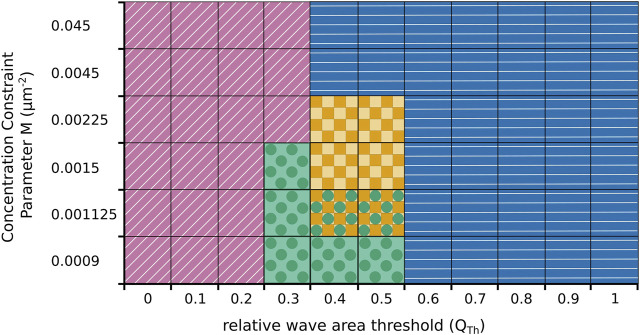
Parameter plane spanned by the threshold value *Q*_*Th*_ in the relative wave area and the concentration constraint parameter *M* that controls regulation of the cortical coverage with the activatory component *c*. Simulations result either in fan-shaped cells (purple oblique lines), in amoeboid cell (blue horizontal lines), or in stationary circular cells (green circles). Only at intermediate parameter values, spontaneous switching between amoeboid and fan-shaped cells is observed (yellow squares). In some cases, two behaviours coexist and the particular dynamics depends on the initial condition (yellow squares and green circles).

We also systematically explored the impact of changes in the model parameters on our findings, see Figure 6 for an illustrative summary. As expected, our numerical simulations revealed that the switching behavior critically depends on the choice of the threshold value *Q*_*Th*_. For low values of *Q*_*Th*_ only fan-shaped cells are observed (marked by purple oblique lines in Figure 6) and for large values cells exclusively move in an amoeboid fashion (marked by blue horizontal lines). Repeated switching between both states only occurs at intermediated *Q*_*Th*_-values around 0.5 (yellow squares). However, whether the switching regime at intermediate *Q*_*Th*_-values is observed or not also depends on the value of the concentration constraint parameter *M* that determines how tightly the cortical *c*-coverage is regulated to match the target value of the amoeboid or fan-shaped state. As can be seen in Figure 6, a tight regulation suppresses the switching regime. In this case, the cell remains locked to the amoeboid or the fan-shaped state, so that for intermediate *Q*_*Th*_-values a sharp boundary between permanently amoeboid and permanently fan-shaped motility is observed. Only for values of *M* below 0.0045, the switching regime emerges (yellow squares). It is, however, also bounded towards lower values of *M* and disappears if *M* is reduced to values below 0.0015. At the same time, a new dynamical regime emerges in this parameter range, where the cell converges to a stationary circular shape, completely filled with a *c*-rich domain (marked by green circles).

## 4 Discussion

Our experimental recordings show that motile *D. discoideum* cells with a deficiency in the RasGAP NF1 spontaneously switch back and forth between amoeboid and fan-shaped modes of motion. This observation suggests that amoeboid and fan-shaped modes can be seen as coexisting behavioral traits of the same cell rather than signatures of heterogeneity in a cell population, where different modes of motility occur in different cells due to cell-to-cell variability. Thus, repeated noise-induced switches between these two states can spontaneously occur in the same cell and do not require changes in the intracellular parameters by drug treatment or altered gene expression to initiate a transition.

This was less obvious from previous experimental observations of the fan-shaped mode, where typically fractions of fan-shaped cells across populations were reported. Examples include *amiB*-null cells (Asano et al., 2004; Asano et al., 2008), axenic wildtype cells developed under low density conditions (Cao et al., 2019b), and, more recently, also knockout cells deficient in the Arp2/3 inactivating factor GmfA (glia maturation factor) (Fujimoto et al., 2022). However, already in these earlier recordings, occasional switches at the single cell level can be seen (Cao et al., 2019b; Fujimoto et al., 2022). We assume that in these cases, the fan-shaped mode is more stable, so that switches within the same cell are less likely to be observed. Similarly, externally induced parameter changes, such as synthetically clamped levels of phosphatidylinositol-4,5-bisphosphate (PtdIns(4,5)P2) and Ras/Rap activity (Miao et al., 2017), or a reduced protrusive strength of the actin cortex by treatment with latrunculin B (Cao et al., 2019b), may result in altered life times of the fan-shaped state.

Recently, the different migratory modes have also been characterized in terms of the traction force patterns that are associated with their intracellular actomyosin distributions, revealing that even within the population of fan-shaped cells, different propulsion mechanisms can be distinguished (Ghabache et al., 2021). Note also that in several of these earlier studies, a third so-called oscillatory mode was reported, where cells adopted a periodically breathing, circular, and almost non-motile configuration (Miao et al., 2017; Cao et al., 2019b; Ghabache et al., 2021). Longer periods of this behavior were not observed in our recordings but can be most likely associated with the stationary circular morphologies observed in our model simulations, see Figure 6 (green circles). In our experiments, stationary circular cells that resemble the oscillatory mode emerged only occasionally and transiently as part of a switching event.

Periodic dynamics reminiscent of wave-mediated switching between amoeboid and fan-shaped modes has been observed already earlier in axenic cells (Gerisch et al., 2011; Gerisch et al., 2012). Here, time intervals without any prominent actin wave activity alternate with transient periods of wave growth and expansion across the entire ventral cortex. In the light of our findings, these episodes can be interpreted as incomplete transitions from amoeboid to fan-shaped mode, where a growing wave eventually occupies the entire bottom cortex but does not succeed in establishing a stable fan. This is in line with the observation that the fan-shaped mode is only rarely seen in axenic wildtype cells under normal culture conditions.

To model the motility of adherent cells, nonlinear reaction-diffusion systems are a widely used choice. They successfully capture the dynamics of cell polarity (Jilkine and Edelstein-Keshet, 2011), which is often related to conservation of some of the participating components (Otsuji et al., 2007; Mori et al., 2008; Bernitt et al., 2017; Halatek et al., 2018), to mutual inhibition (Matsuoka and Ueda, 2018), or to external chemical gradients (Beta et al., 2008; Iglesias and Devreotes, 2008). In combination with an auxiliary phase field to account for the moving cell boundary, this method was first applied to keratocyte motility (Shao et al., 2010; Shao et al., 2012; Ziebert et al., 2012; Camley et al., 2017) and later extended to neutrophil and *Dictyostelium* morphodynamics (Najem and Grant, 2013; Moure and Gomez, 2016; Alonso et al., 2018; Imoto et al., 2021). Here, dynamic wave patterns in the intracellular reaction-diffusion system drive the formation of membrane protrusions to propagate the cell forward. Also, the interactions among adjacent cells and solid boundaries were incorporated into this modeling framework (Löber et al., 2015; Kulawiak et al., 2016; Moreno et al., 2022), as well as extensions to three dimensions (Cao et al., 2019a; Winkler et al., 2019), corresponding to locomotion in enclosed environments (Nagel et al., 2014).

Several models were proposed that exhibit both amoeboid and fan-shaped modes, including a lattice model at the level of the actin dynamics (Nishimura et al., 2009), a level set approach relying on a biased excitable signaling network (Miao et al., 2017), as well as phase field models that focus on nonlinear signaling kinetics (Moreno et al., 2020) or incorporate mechanochemical coupling and force generation (Cao et al., 2019b; Ghabache et al., 2021). Also much simpler models of adhesive vesicles with curved membrane proteins exhibit similar shape transitions (Sadhu et al., 2021). To account for our experimental observations, we extended an established motility model by incorporating the coexistence of two stable modes of locomotion (bistability). We chose a phenomenological model that was designed to explain the cell-to-cell variability in the motion patterns of motile *D. discoideum* cells (Alonso et al., 2018) and that was later found to exhibit both amoeboid and fan-shaped modes of locomotion (Moreno et al., 2020). This model is based on a noisy bistable reaction-diffusion system to mimic the intracellular dynamics, combined with a dynamic phase field to take the cell shape dynamics into account. It has also been extended to a two-variable activator-inhibitor version to describe wave-driven cytofission events (Flemming et al., 2020).

In the fan-shaped state, most of the substrate-attached bottom membrane is covered by a composite PIP_3_/actin wave, whereas in the amoeboid state, only smaller patches of activity are observed along the cell border. Consequently, we included an additional bistability into the model that allowed for a noise-induced switching between an amoeboid state with low coverage of the activatory component *c*, and a fan-shaped state, where a large fraction of the cell area is covered with *c*. In this way, we could qualitatively reproduce our experimental findings. Furthermore, our modeling study predicted that spontaneous switching between the two modes is only observed in a limited window of intermediate values of the model parameters *Q*_*Th*_ and *M*, see Figure 6. Even though these parameters cannot be unambiguously linked to experimentally accessible quantities, we may rationalize their meaning in the light of the current state of our knowledge. Fan-shaped cells occur under conditions, where the concentration of typical cell front markers, such as PIP_3_ or active Ras, is upregulated, e.g., by synthetically clamping the intracellular PtdIns(4,5)P2 at low or the Ras/Rap activity at high levels (Miao et al., 2017). In our model, the threshold parameter *Q*_*Th*_ sets the probability that the cell will adopt such a state of high front marker levels: The lower the value of *Q*_*Th*_ the higher the probability that the cell will converge to a fan-shaped state. For this reason, regular switching between amoeboid and fan-shaped modes can only occur at intermediate values of *Q*_*Th*_. Altering the value of *Q*_*Th*_ in the model thus corresponds to an experiment, where the probability to switch to the fan-shaped mode is gradually changed. This can be potentially tested in future dosing experiments that monitor the switching rate as a function of intracellular PIP_3_ or Ras levels once a technique becomes available that allows for a quantitative tuning of such intracellular signaling activities.

The concentration constraint parameter *M*, on the other hand, controls how tightly the cell regulates the intracellular signaling levels to the target values that correspond to the amoeboid or fan-shaped states. For large values of *M* there is little chance to escape from an established amoeboid or fan-shaped state, so that only for sufficiently low values of *M* switches may occur. It remains unclear how this parameter can be directly changed in experiments. But the characteristic time scale of this regulatory process that is controlled by the parameter *M* could be assessed by measuring the response time of basal actin waves to changes in intracellular PIP_3_ or Ras levels. Again, a quantitative dosing technique, which is currently not available in sufficient precision, would be required to estimate *M* from such experiments. But we are optimistic that future developments in optogenetic tools will provide the necessary means to establish a more quantitative link between experimentally accessible quantities and modeling parameters.

## Data Availability

The raw data supporting the conclusions of this article will be made available by the authors, without undue reservation.

## Supplementary material

The Supplementary Material for this article can be found online at: https://www.frontiersin.org/articles/10.3389/fcell.2022.898351/full#supplementary-material

Supplementary Presentation 1 Supplementary Material (figures and tables).

Supplementary Presentation 2 Appendix.
