# Supplementary material for: Spontaneous transitions between amoeboid and keratocyte-like modes of migration
Source: Front Cell Dev Biol. 2022 Sep 30;10:898351. doi: 10.3389/fcell.2022.898351 (PMC9563996; doi:10.3389/fcell.2022.898351)
Supplement: Supplementary file 3 [file Presentation1.pdf]

Supplementary Material

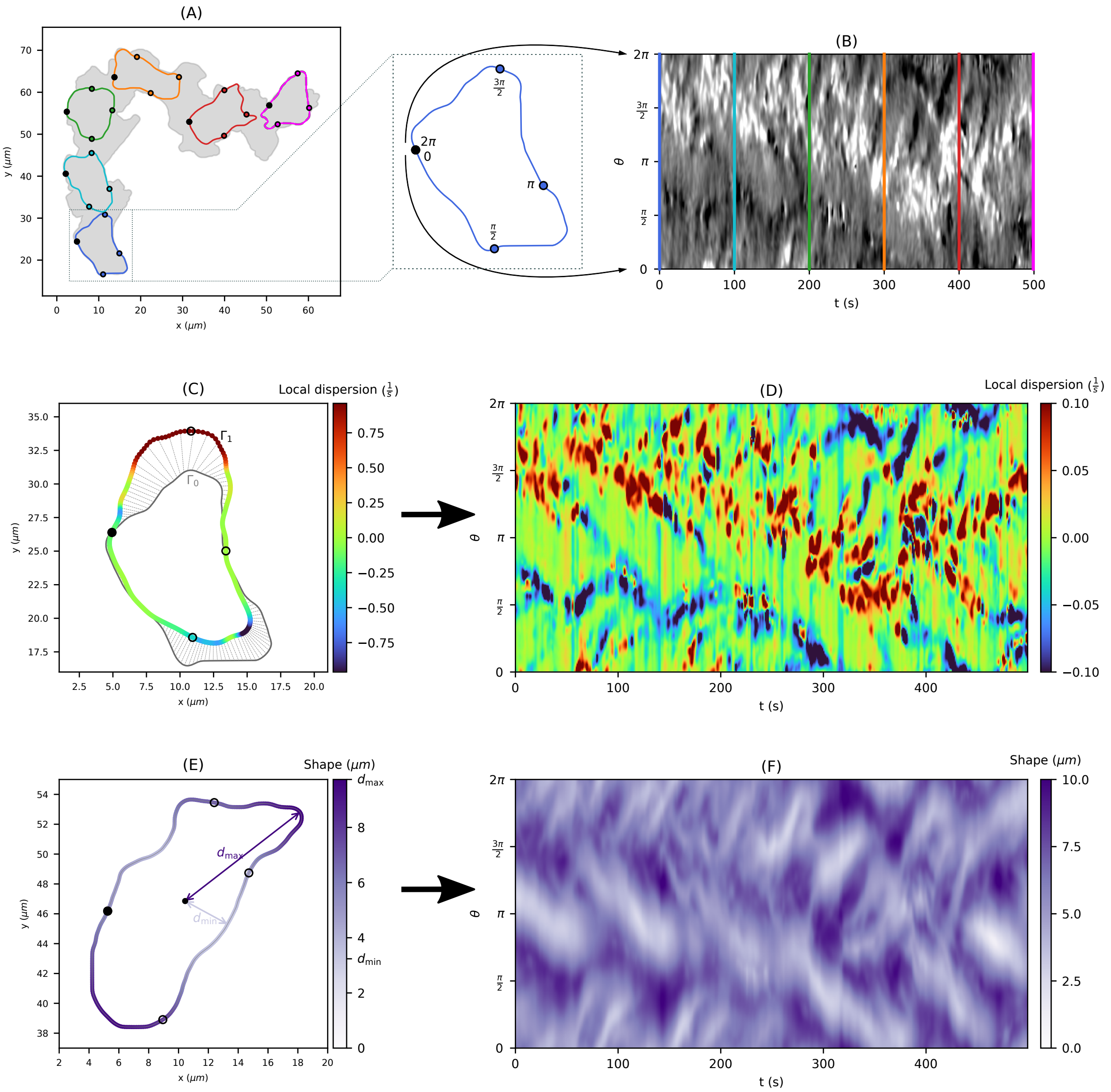

Figure S1. Computation of geometric and dynamic quantities of a motile cell and their representation as kymographs. (A) Cell track with underlying spatio-temporal coordinate system shown for exemplary contours and virtual markers: $\theta = 0$ (black filled circle) and $\theta \in \{\frac{\pi}{2}, \pi, \frac{3\pi}{2}\}$ (empty circle). (B) A quantity of interest (the example here is the local motion) can be displayed as a two-dimensional kymograph representation, with the time coordinate on the x-axis and the normalized arc length coordinate $\theta \in [0, 2\pi)$ on the y-axis (colored lines representing single cell contours). (C) Local dispersion showing regions of virtual marker thinning (expansions, red), virtual marker clustering (retractions, blue), and neutral regions (green). (E) Distances from virtual markers on the contour to the center of mass of the cell (so-call contour shape, color-coded in purple). (D, F) Corresponding kymographs showing local dispersion and distances to the center of mass as a function of time and space.

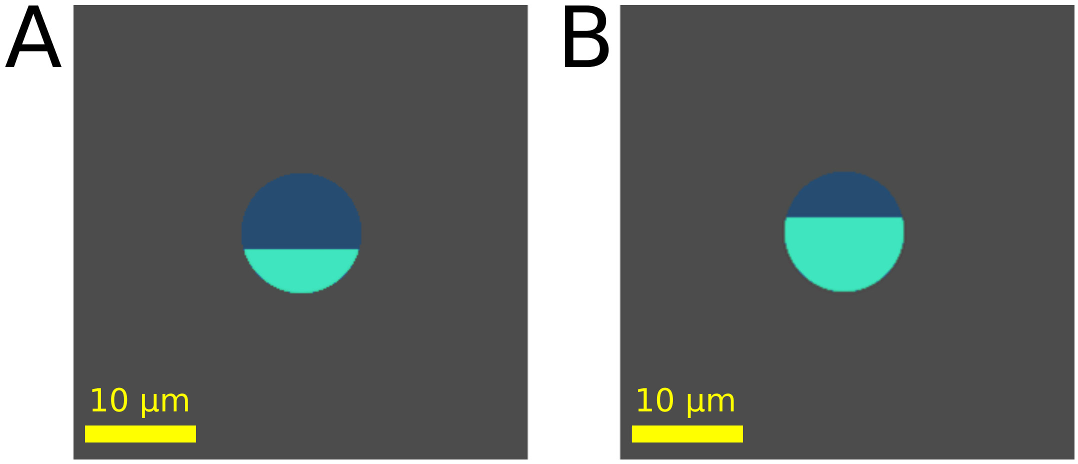

Figure S2. Initial conditions for the model simulations. All simulations, which have been taken into account for the analysis in this article, started with either 25% (A) or 75% (B) of the cell area covered by high values of the effective force generating component c. After starting the simulations, the cell shape and the intracellular distribution of c randomly evolved according to the stochastic model equations, so that correlations with the initial cell shape and c distribution were rapidly lost, see also the corresponding movie.

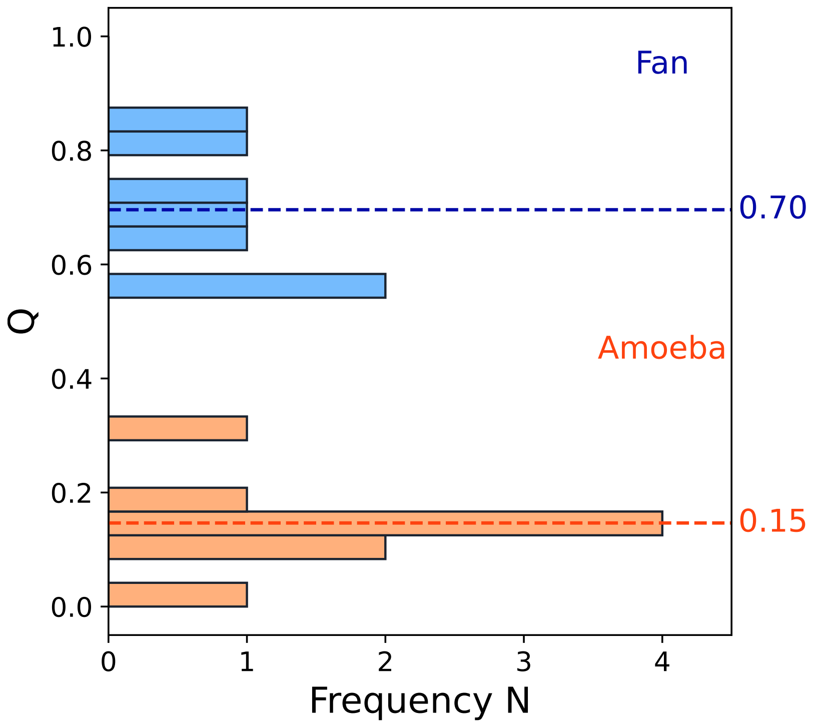

Figure S3. Histogram of Q values generated for experimentally recorded cells taken from Ghabache et al. (2021) and Miao et al. (2017), see details below. All cells were recorded by fluorescence microscopy. The blue bars represent cells classified in the corresponding papers as fan-shaped cells. The dark blue dashed line is the mean value $Q = 0.70$ of all fan-shaped cells. The orange bars represent cells classified in the corresponding papers as amoeboid cells. The dark orange dashed line is the mean value $Q = 0.15$ of all amoeboid cells. The fact that all Q values of amoeboid cells are below $Q = 0.50$ and all Q values of fan-shape cells are above $Q = 0.50$ support our classification of amoeboid and fan-shape cells based on the value of Q.

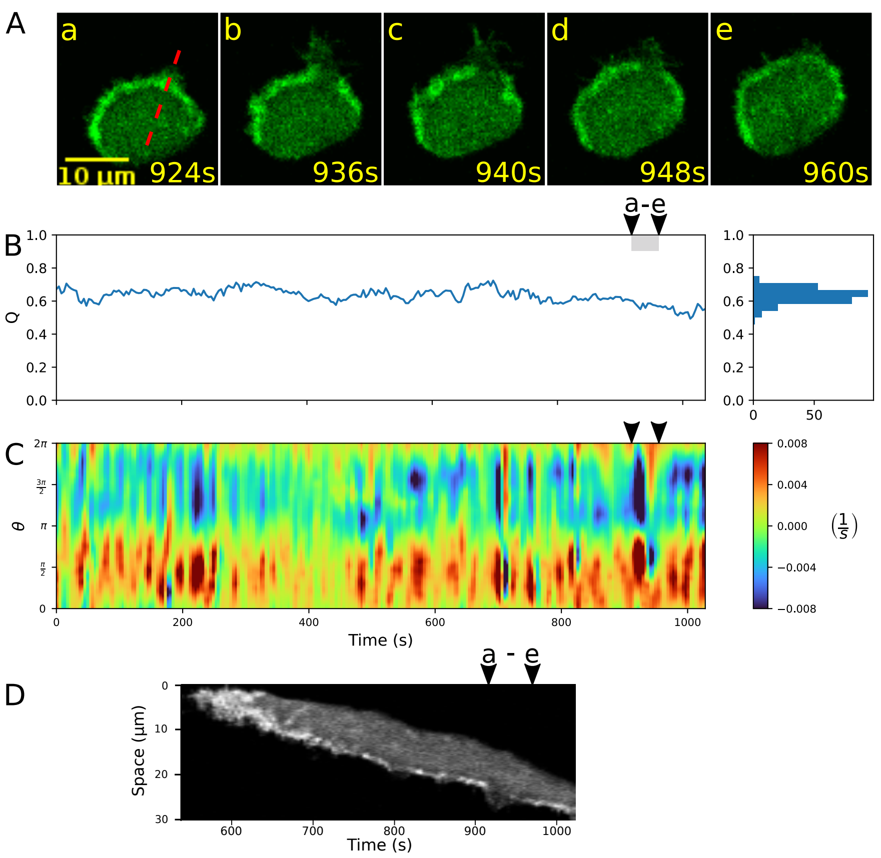

Figure S4. Small protrusions at the leading edge of fan-shaped cell. (A) Snapshots displaying growth and decay of a protrusion at the leading edge (a) to (e). (B) Time evolution of the relative wave area Q. The shaded area in between (a) and (e) indicates the episode of protrusion formation. Histogram on the right-hand side shows the frequency of Q-values. (C) Kymograph of the local dispersion taken along the cell contour. (D) Fluorescence kymograph taken along the dashed red line displayed in the first panel of (A). Arrowheads above the kymographs mark the time points of the corresponding fluorescence images in (A).

Small protrusions at the leading edge of fan-shaped cells

At the leading edge of persistently moving fan-shaped cells, we repeatedly observed the emergence of small, short-lived protrusions. In Fig. S4A, the formation and decay of such a protrusion is illustrated in a sequence of snapshots (a)–(e) from the fluorescence recording of a stable fan-shaped cell. The entire event extends over a short time window of about 36 s, which is typical for the life-time of these structures. Due to their small size, their formation does not affect the overall value of the relative wave area Q, see Fig. S4B, where Q remains around 0.6 for the entire measurement time. However, in the local dispersion kymograph of the cell contour in panel C, protrusion formation can be clearly detected as a strongly localized, extending region (red), with a simultaneous increased retraction along the rest of the contour (blue), see the time period between (a) and (e), as well as the time period shortly after $t = 200$ s.

The growing protrusion is accompanied by a decrease of the fluorescence intensity in its vicinity. In particular, the front of the actin wave that pushes the cell forward is directly affected by the growing protrusion, such that the leading wave segment is disrupted, indicating a depletion of F-actin, which causes a breakup of the wave segment at the position of protrusion formation, see Fig. S4A at time frames (b)–(d). We thus conclude that the growing protrusion competes with the wave for the common pool of

actin, consuming some of it in its surrounding in order to push the plasma membrane outwards. When the protrusion has decayed, the disrupted actin segment at the wave front heals and recovers its initial fluorescence intensity. This can be also seen in the fluorescence intensity kymograph in Fig. S4D, taken along the red dashed line displayed in the first snapshot of panel A.

Small protrusions at the leading edge of fan-shaped cells have been noted earlier (Flemming et al., 2020) and can be also induced by chemotactic cues (Ecke and Gerisch, 2019). However, based on the limited amount of available data, their precise nature remains elusive. They resemble small, short-lived pseudopods that are commonly observed in *D. discoideum* cells when moving in an amoeboid fashion (Swanson and Taylor, 1982; Rubino et al., 1984; Wessels et al., 1988) but it is also conceivable that their formation is driven by a blebbing mechanism (Tyson et al., 2014).

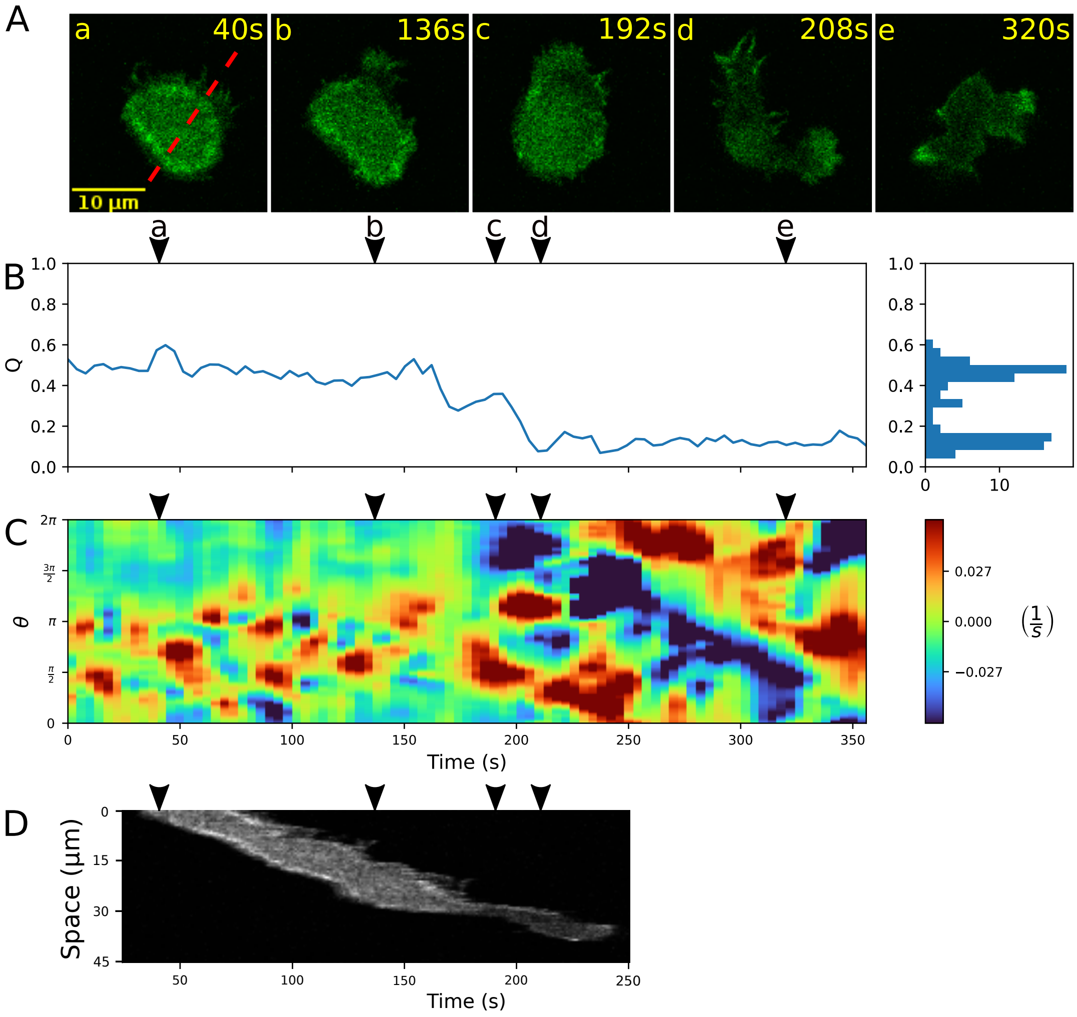

Figure S5. Breakdown of actin wave and simultaneous switching from fan-shaped to amoeboid mode of migration. (A) Snapshots showing a cell in moving fan-shaped mode pushed by a stable actin wave (a, b). The breadown of the actin wave and the switching is visible in (c) to (e) , and the cell moving in amoeboid mode of motion in (e). (B) Time evolution of the relative wave area Q. The arrowheads indicate the time points of the snapshots in (A). Histogram on the right-hand side shows the frequency of Q-values. (C) Kymograph of the local dispersion taken along the cell contour. (D) Fluorescence kymograph taken along the dashed red line indicated in the first panel of (A).

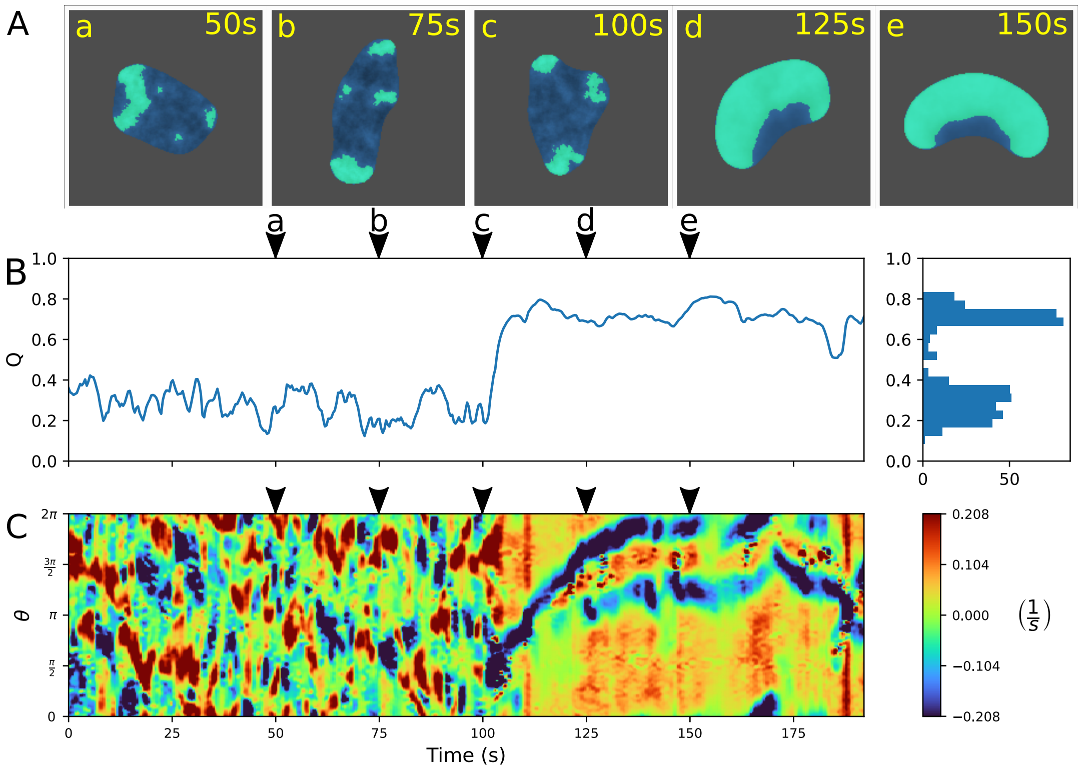

Figure S6. Switching from amoeboid motion to fan-shaped migration mode in model simulations. (A) Snapshots of amoeboid migration mode (a, b, c) switching to fan-shaped migration (d, e). (B) Depiction of relative wave area $Q = A_W/A_C$, where at the left half, low values with high variations indicate the amoeboid migration, while at the right half high values of Q with low variations are associated with fan-shaped motion. (C) Local dispersion kymograph along the cell contour. The arrowheads with labels (a) to (e) mark the time points corresponding to the snapshots in (A). The concentration constraint parameter was set to $M = 0.00225$ and the threshold value to $Q_{Th} = 0.5$; for all other model parameters see Table S1.

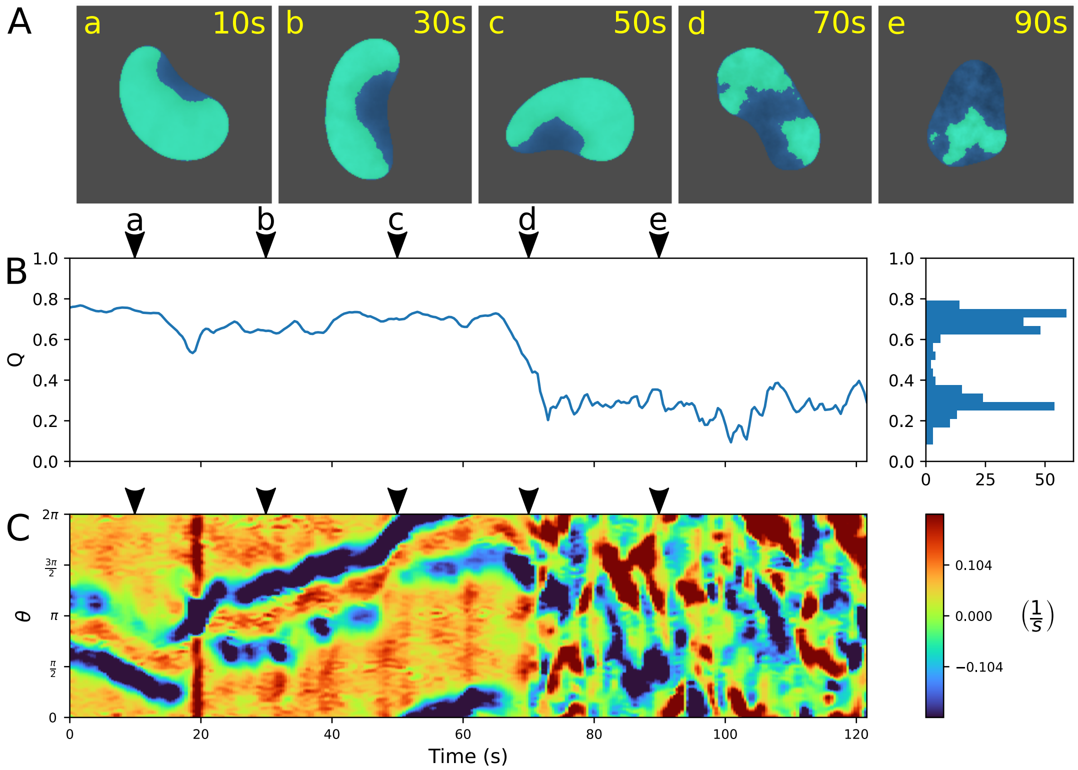

Figure S7. Model simulation of wave breakdown and associated motility mode switching from fan-shaped to amoeboid motion. (A) Snapshots of fan-shaped mode (a, b, c) switching to amoeboid motility (d, e). (B) Time trace of relative wave area $Q = A_W/A_C$. In the first half, high values with low variations are associated with the fan-shaped migration mode, while in the second half, low values with stronger variations indicate the amoeboid motion. (C) Local dispersion kymograph along the cell contour. The arrowheads with labels (a) to (e) mark the time points corresponding to the snapshots in (A). The concentration constraint parameter was set to $M = 0.0015$ and the threshold value to $Q_{Th} = 0.5$; for all other model parameters see Table S1.

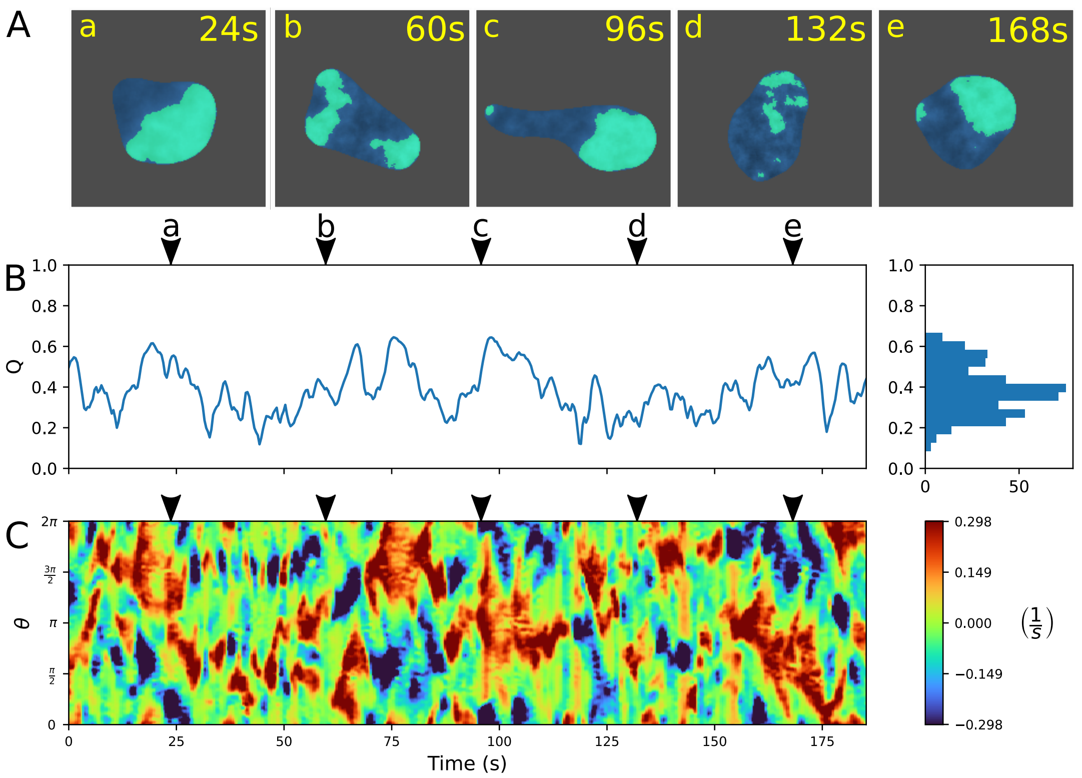

Figure S8. Model simulation of failed attempt to switch migration mode from amoeboid to fan-shaped. (A) Snapshots of growing and decaying waves which did not switch to a stable fan-shaped configuration. (B) Depiction of relative wave area $Q = A_W/A_C$. The strongly fluctuating Q value with a mean around 0.4 is reflecting the repeatedly growing waves which did not archive stability. (C) Local dispersion kymograph along the cell contour. The arrowheads with labels (a) to (e) mark the time points corresponding to the snapshots in (A). The concentration constraint parameter was set to $M = 0.0045$ and the threshold value to $Q_{Th} = 0.4$; for all other model parameters see Table S1.

Analysis of the distance from the contour to the center of the cell

Our model was not specifically tuned to reproduce geometrical features of the experimentally observed cell shapes. Thus, the distance from the center of the cell to the contour provides an additional opportunity to compare experimental observations and simulation results, i.e., an independent quality check for the performance of our model.

In order to interpret the data, let us first consider what we expect for the different cases. During amoeboid migration without any external cue, pseudopod activity is randomly distributed all around the cell contour. Hence, on a time scale much longer than the typical pseudopod lifetime, the distance from the center to the contour of an amoeboid cell will evolve randomly and will, when averaged over time, approach half of the cell diameter for each point on the contour (a typical cell diameter is about 10μm). The mean distance to center for amoeboid contours will thus converge to a flat profile at a distance of around 5μm, while the histogram of the center to contour distances for all individual contour positions will be symmetrically distributed around this value. This is indeed observed both in the experimental (Fig. S9) and in the numerical data (Fig. S10). When considering time intervals, during which the cell moves in an amoeboid fashion (highlighted in yellow in the kymographs (B) in Figs. S9 and S10), we see in the experimental as well as in the simulation data no distinct coherent patterns. Figures S9 (C) and S10 (C) depict the distance from the center to the contour for all individual contours (grey lines) in the highlighted time interval, and the black line is the average over all these contours, displaying an almost constant value of around 5μm. Figures S9 (D) and S10 (D) show the histograms for all contour positions during the highlighted time interval, which almost symmetrically center around the mean value.

In the case of fan-shaped motion, the cell adopts a kidney-like shape, elongated perpendicular to the direction of motion. Thus, the front and rear parts of the cell border are closer to the center compared the left and right parts. This shape is stably maintained while the cell is moving. Hence, we expect to see a characteristic pattern in the distances of the contours from the center for these cells. This is indeed observed during time intervals, when cells move in the fan-shaped mode, see the time interval highlighted in yellow in the kymographs (G) in Figs. S9 and S10. Here, the kidney shape is reflected by an alternating pattern of dark and bright stripes. This is also visible in the plots of the individual contours from the highlighted time interval (grey lines) and in their average (black line) in Figs. S9 (H) and S10 (H). As the kidney shape persists over time, the histograms of the distances taken over the individual contours display a characteristic asymmetric distribution with three local maxima, which is, however, more clearly seen in the numerical than in the experimental data, see Figs. S9 (I) and S10 (I).

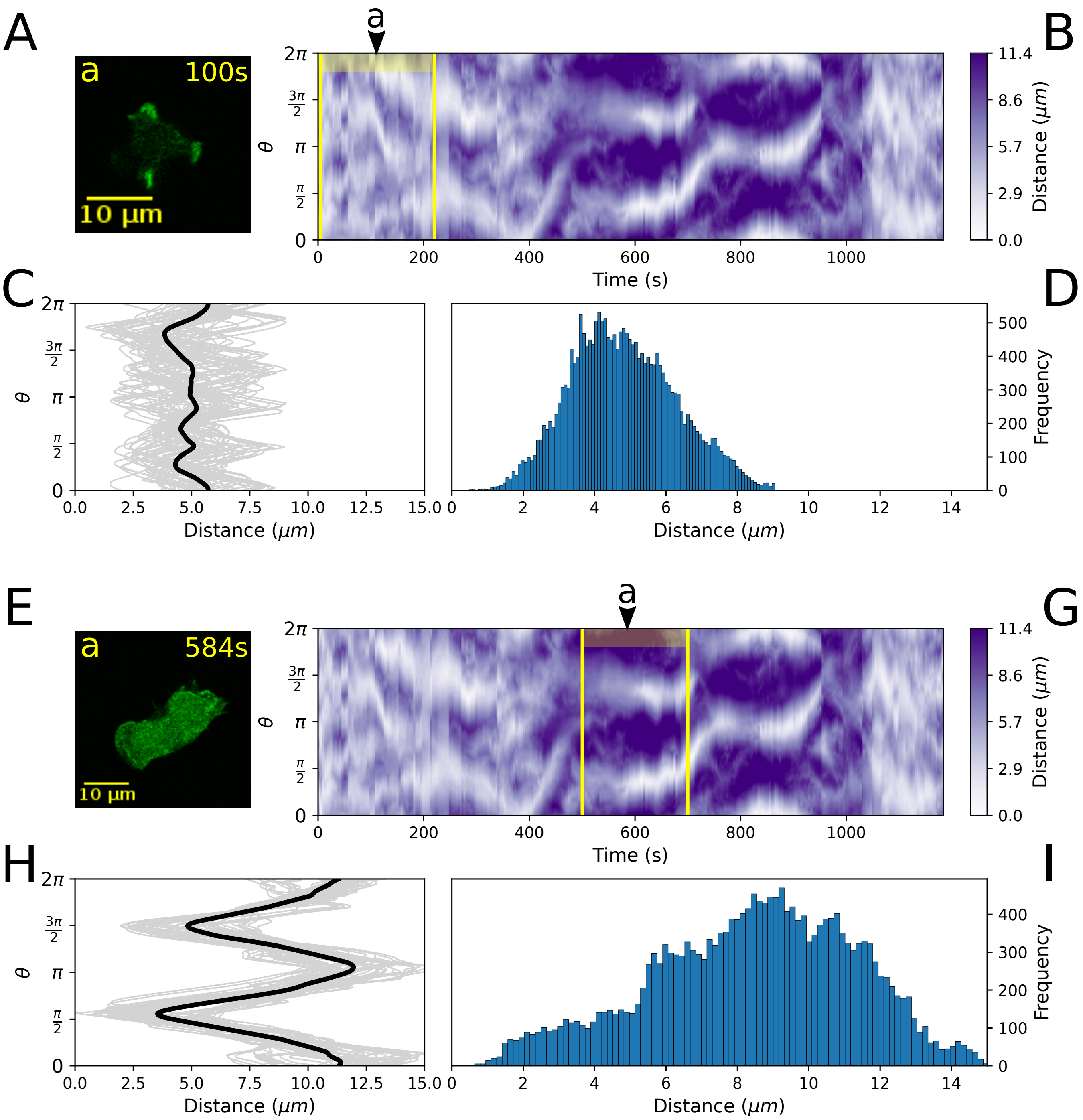

Figure S9. Illustration of the cell center to contour distance for an experimentally observed cell while moving in the amoeboid (A – D) and in the fan-shaped mode (E – I). (A, E) Snapshots of the cell, taken by fluorescence microscopy, while migrating in amoeboid (A) and fan-shape mode (E). (B, G) Kymograph of the cell center to contour distance, illustrating the distance of each position on the contour (0 to π) over time. White indicates small and dark blue large distances of a contour point from the center. The arrowheads (a) mark the corresponding time points of the cell shown in the snapshots (A) and (E), respectively. For further explanations of the kymograph concept see Fig. S1. (C, H) Distance to center plot of each individual contour (gray lines) in the time interval highlighted in yellow in (B, G). The black line is the average of all contours in the highlighted time interval. (D, I) Histogram of the distance to the center for all positions on the contour over the highlighted time interval.

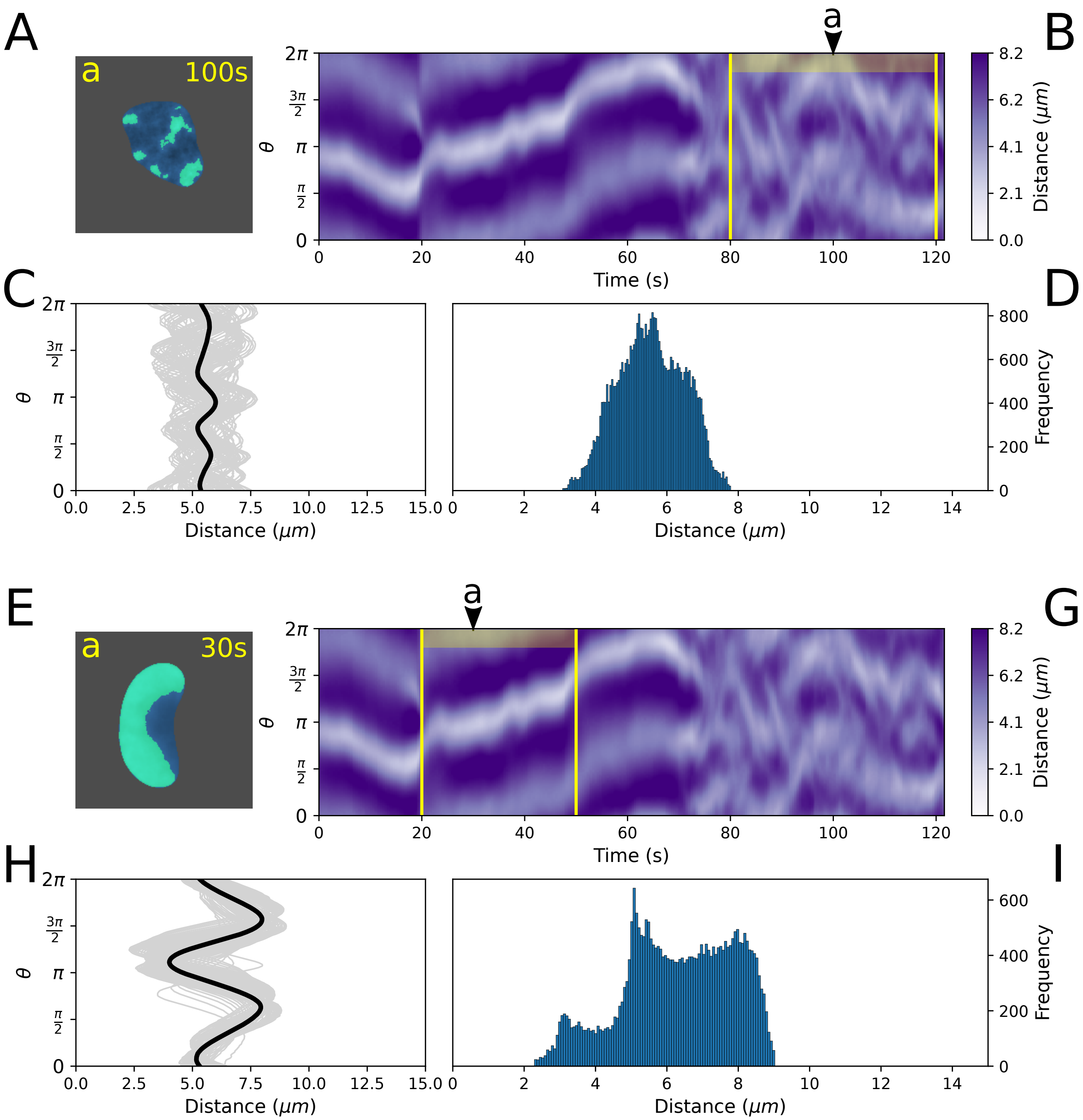

Figure S10. Illustration of the cell center to contour distance for a numerically simulated cell while moving in the amoeboid (A – D) and in the fan-shaped mode (E – I). (A, E) Snapshots of the cell while migrating in amoeboid (A) and fan-shape mode (E). (B, G) Kymograph of the cell center to contour distance, illustrating the distance of each position on the contour (0 to π) over time. White indicates small and dark blue large distances of a contour point from the center. The arrowheads (a) mark the corresponding time points of the cell shown in the snapshots (A) and (E), respectively. For further explanations of the kymograph concept see Fig. S1. (C, H) Distance to center plot of each individual contour (gray lines) in the time interval highlighted in yellow in (B, G). The black line is the average of all contours in the highlighted time interval. (D, I) Histogram of the distance to the center for all positions on the contour over the highlighted time interval.

Video 1: Video of a fluorescent *D. discoideum* DdB NF1 KO cell, corresponding to Figure 2 . Repeated switching between amoeboid and fan-shaped modes of migration. The frame rate is 4 seconds. F-actin is labeled with Lifeact-GFP. Total time of video 1800 seconds.

Video 2: Video of a fluorescent *D. discoideum* DdB NF1 KO cell, corresponding to Figure 3. Growth and decay of an actin wave is shown, that does not stabilize into a fan-shape mode. The frame rate is 4 seconds. F-actin is labeled with Lifeact-GFP. Total time 1188 seconds.

Video 3: Video of a fluorescent *D. discoideum* DdB NF1 KO cell, corresponding to Figure 4. A cell switching from amoeboid motion into the fan-shaped mode. The frame rate is 4 seconds. F-actin is labeled with Lifeact-GFP. Total time 1752 seconds.

Video 4: Model simulation video, showing repeated noise-induced switching between amoeboid and fan-shaped mode, corresponding to Figure 5.

Video 5: Model simulation video, showing the initial conditions for the model simulations, corresponding to Figure S2.

Video 6: Video of a fluorescent *D. discoideum* DdB NF1 KO cell, corresponding to Figure S4. The growth and decay of a small protrusion at the leading edge of a fan-shaped cell. The frame rate is 4 seconds. F-actin is labeled with Lifeact-GFP. Total time 104 seconds.

Video 7: Video of a fluorescent *D. discoideum* DdB NF1 KO cell, corresponding to Figure S5. Breakdown of the actin wave and the associated switching from fan-shaped migration to amoeboid motion. The frame rate is 4 seconds. F-actin is labeled with Lifeact-GFP. Total time 360 seconds.

Video 8: Model simulation video, showing switching from amoeboid to fan-shaped motion, corresponding to Figure S6.

Video 9: Model simulation video, showing breakdown of the wave and switching from fan-shaped mode to amoeboid locomotion, corresponding to Figure S7.

Video 10: Model simulation video, showing several failed attempts to switch migration mode, corresponding to Figure S8.

Figure S3: Cells used for the analysis displayed in Fig. S3 were taken from Ghabache et al. (2021) and Miao et al. (2017). All cells were shown in the main figures of the articles, except two of them that were part of the supplementary material. The analysis was performed on the fluorescence movies corresponding to the cells shown in the figures. All movies were provided in the supplementary material of the articles.

Cells classified as fan-shape cells in Ghabache et al. (2021): Fig:2A (left side) - Movie EV1, Fig:2A (right side) - Movie EV2, Fig:2C (left side) - Movie EV1, and Fig:2C (right side) - Movie EV2.

Cells classified as fan-shape cells in Miao et al. (2017): Fig:2D (right side) - Movie 41556 2017 BFncb3495 MOESM69 ESM, Fig:4A (middle) - Movie 41556 2017 BFncb3495 MOESM69 ESM, Fig:4A (middle) -Movie 41556 2017 BFncb3495 MOESM73 ESM, and Fig:4D (rigth side) 41556 2017 BFncb3495 MOESM74 ESM.

Cells classified as amoeboid cells in Ghabache et al. (2021): Fig:3A - Movie EV5, Fig:4A - Movie EV7, Movie EV6, and Movie EV8.

Cells classified as amoeboid cells in Miao et al. (2017): Fig:2A (right side) - Movie 41556 2017 BFncb3495 MOESM68 ESM, Fig:2A (left side) - Movie 41556 2017 BFncb3495 MOESM68 ESM, Fig:2B (top left side) - Movie 41556 2017 BFncb3495 MOESM69 ESM, Fig:4A (left side) - Movie 41556 2017 BFncb3495 MOESM73 ESM, and Fig:4B (top left side) - Movie 41556 2017 BFncb3495 MOESM74 ESM.

Table S1. Parameter values for the numerical model.

Parameter	Value	Units	Meaning	Reference
D	0.5	$\mu m^2/s$	Diffusion coefficient	(Alonso et al., 2018)
k_a	2	s^{-1}	Reaction rate	(Alonso et al., 2018)
ρ	0.02	s^{-1}	Degradation rate	(Moreno et al., 2020)
σ	0.15	s^{-2}	Noise strength	(Alonso et al., 2018)
τ	2	$pNs\,\mu m^{-2}$	Membrane dynamics time-scale	(Shao et al., 2010)
				& (Ziebert et al., 2012)
γ	2	pN	Surface tension	(Shao et al., 2010)
ϵ	0.75	μm	Membrane thickness	(Shao et al., 2010)
β	22.22	$pN\mu m^{-3}$	Parameter for total area constraint	(Alonso et al., 2018)
A_0	113	μm^2	Area of the cell	(Alonso et al., 2018)
δ_0	0.5	-	Bistability critical parameter	(Alonso et al., 2018)
k_η	0.1	s^{-1}	Ornstein-Uhlenbeck rate	(Alonso et al., 2018)
α	3	$pN\mu m^{-1}$	Active tension	(Ziebert et al., 2012)
β_C	0.022	$pN\mu m^{-5}$	Control parameter for total area constraint	
τ_β	0.2	s	Time-scale for area constraint	
τ_δ	0.2	s	Time scale for bistability condition	

REFERENCES

Alonso, S., Stange, M., and Beta, C. (2018). Modeling random crawling, membrane deformation and intracellular polarity of motile amoeboid cells. PLOS One 13, e0201977. doi:10.1371/journal.pone.0201977

Ecke, M. and Gerisch, G. (2019). Co-existence of Ras activation in a chemotactic signal transduction pathway and in an autonomous wave - forming system. Small GTPases 10, 72–80. doi:10.1080/21541248.2016.1268666

Flemming, S., Font, F., Alonso, S., and Beta, C. (2020). How cortical waves drive fission of motile cells. Proceedings of the National Academy of Sciences 117, 6330–6338. doi:10.1073/pnas.1912428117

Ghabache, E., Cao, Y., Miao, Y., Groisman, A., Devreotes, P. N., and Rappel, W.-J. (2021). Coupling traction force patterns and actomyosin wave dynamics reveals mechanics of cell motion. Molecular Systems Biology 17, e10505. doi:10.15252/msb.202110505

Miao, Y., Bhattacharya, S., Edwards, M., Cai, H., Inoue, T., Iglesias, P. A., et al. (2017). Altering the threshold of an excitable signal transduction network changes cell migratory modes. Nature Cell Biology 19, 329–340. doi:10.1038/ncb3495

Moreno, E., Flemming, S., Font, F., Holschneider, M., Beta, C., and Alonso, S. (2020). Modeling cell crawling strategies with a bistable model: From amoeboid to fan-shaped cell motion. Physica D: Nonlinear Phenomena 412, 132591. doi:10.1016/j.physd.2020.132591

Rubino, S., Fighetti, M., Unger, E., and Cappuccinelli, P. (1984). Location of actin, myosin, and microtubular structures during directed locomotion of Dictyostelium amebae. Journal of Cell Biology 98, 382–390. doi:10.1083/jcb.98.2.382

Shao, D., Rappel, W.-J., and Levine, H. (2010). Computational Model for Cell Morphodynamics. Physical Review Letters 105, 108104. doi:10.1103/PhysRevLett.105.108104

Swanson, J. A. and Taylor, D. L. (1982). Local and spatially coordinated movements in Dictyostelium discoideum amoebae during chemotaxis. Cell 28, 225–232. doi:10.1016/0092-8674(82)90340-3

Tyson, R. A., Zatulovskiy, E., Kay, R. R., and Bretschneider, T. (2014). How blebs and pseudopods cooperate during chemotaxis. Proceedings of the National Academy of Sciences 111, 11703–11708. doi:10.1073/pnas.1322291111

Wessels, D., Soll, D. R., Knecht, D., Loomis, W. F., De Lozanne, A., and Spudich, J. (1988). Cell motility and chemotaxis in Dictyostelium amebae lacking myosin heavy chain. Developmental Biology 128, 164–177. doi:10.1016/0012-1606(88)90279-5

Ziebert, F., Swaminathan, S., and Aranson, I. S. (2012). Model for self-polarization and motility of keratocyte fragments. Journal of The Royal Society Interface 9, 1084–1092. doi:10.1098/rsif.2011.0433
