# Supplementary material for: Spontaneous transitions between amoeboid and keratocyte-like modes of migration
Source: Front Cell Dev Biol. 2022 Sep 30;10:898351. doi: 10.3389/fcell.2022.898351 (PMC9563996; doi:10.3389/fcell.2022.898351)
Supplement: Supplementary file 9 [file Presentation2.pdf]

Appendix

Motile cells are complex and highly dynamic. A fundamental challenge in the modeling of entire motile cells is the fact that, on a molecular level, many hundreds of interacting species are involved. They are connected by a network of biochemical reactions and, in the case of cytoskeletal and membrane components, also contribute to the mechanics of the movement process. While parts of this network have been studied in detail, such as, for example, the role of Ras and phosphoinositide signaling or the treadmilling dynamics of actin filaments, other components are only partly explored or not known at all (Devreotes et al., 2017). To date, a detailed mechanistic whole-cell motility model is therefore out of reach.

For this reason, many modeling efforts rely on phenomenological concepts that are not rooted in the details of the underlying molecular mechanisms. They are built on a small number of fundamental physical properties to account for the observed macroscopic behavior. Here, a widely used approach are reaction-diffusion models in combination with a dynamic phase field to describe the cell boundary (Shao et al., 2010; Aranson, 2016). Instead of a detailed mechanical model of the cell membrane and cortex, the phase field takes only the most basic physical properties into account that govern the dynamics of the cell border, namely, interfacial tension, volume conservation, and the presence of active forces that are locally exerted by the cytoskeletal activity. Similarly, the vast network of interacting intracellular components and their subcellular transport is approximated by a small set of reacting and diffusing species (reaction-diffusion system) that captures the observed patterns of intracellular activity. In most cases, these species do not correspond to a single molecular player. They are effective lumped variables that represent an entire set of molecular components, such as, for example, typical cell front markers that trigger protrusive activity at the leading edge (active Ras, PIP_3, freshly polymerized actin). They are coupled to the active tension in the phase field, so that cell shape changes may occur as a consequence of the intracellular dynamics. Despite the obvious conceptional shortcomings of such simplistic models, they serve an important purpose. They reveal the basic dynamical properties of the system and cast them into the form of mathematical equations, which can be analyzed numerically and, in some cases, even analytically to provide results that can be challenged by future experiments.

Numerous research groups have successfully used this approach to describe the motility of different cell types, such as keratocytes, neutrophils, and *D. discoideum* cells (Shao et al., 2012; Najem and Grant, 2013; Imoto et al., 2021). Also the collective behavior of ensembles of many moving cells (Löber et al., 2015; Moure and Gomez, 2019) and their interactions with soft substrates, solid boundaries, and microstructures have been studied using such phenomenological phase field models (Löber et al., 2014; Kulawiak et al., 2016; Honda et al., 2021). While three-dimensional versions of such models became recently available (Cao et al., 2019; Winkler et al., 2019), most phase field models to date are designed to describe two-dimensional projections of a cell.

We have recently proposed a phase field model to account for cell shape changes and locomotion of *D. discoideum* cells to address questions of cell crawling strategies and cell-to-cell variability (Alonso et al., 2018; Moreno et al., 2020). Here, we propose an extension of this model to account for the experimental observations reported in this article. Similar to earlier models by others, we consider the following phase field equation for the cell shape,

$$\tau \frac{\partial \phi}{\partial t} = \gamma \left(\nabla^2 \phi - \frac{G'(\phi)}{\epsilon^2} \right) - \beta \left| \nabla \phi \right| + \alpha \, \phi \, c \left| \nabla \phi \right| , \tag{A1}$$

where $\phi = 1$ denotes the interior of the cell, $\phi = 0$ the exterior, and τ the characteristic time scale of the contour dynamics. The three terms on the right-hand side of Eq. (A1) represent surface tension, area conservation, and active tension, respectively, and will be briefly discussed in the following.

The first term ensures that, due to the membrane tension γ, the cell shape will converge to a circle if no other forces are applied. Here, the function $G(\phi)$ is a double well potential that defines the inside ($\phi = 1$) and the outside ($\phi = 0$) of the cell as the two stable states of the phase field variable. We use $G(\phi) = 18\phi^2(1-\phi)^2$, for details see Alonso et al. (2018). The parameter ϵ controls the width of the transition zone between the inside and outside, i.e., the width of the cell border.

The second term enforces a constant cell volume, i.e., a constant cell area in the case of a two-dimensional projection. In our present simulations, the area conservation was modified compared to the earlier versions of our model. Previously, the cell area was kept constant at all times. However, in our experiments, we recorded a cross-sectional cell area that may strongly fluctuate due to three-dimensional deformations of the cell body. For this reason we adapted the area conservation term to allow for fluctuations of the cell area around an average value A_0 with a characteristic time scale τ_β,

$$\tau_\beta \frac{\partial \beta}{\partial t} = -\beta + \beta_c \left(\int \phi \, dA - A_0 \right) , \tag{A2}$$

where the parameter β_c determines how tightly the cell area is regulated to match the average value A_0. In the limit $\tau_\beta \to 0$, we recover the area conservation term of our previous model (Alonso et al., 2018; Moreno et al., 2020).

As a consequence of intracellular processes, in particular dynamical rearrangements of the actin cytoskeleton, the cell may generate active tension that deforms the cell contour to form, for example, pseudopodia. These active tensions are represented by the third term on the right-hand side of Eq. (A1). The amplitude of the active tension is set by the parameter α, and the intracellular components that actively drive membrane deformations are represented by the concentration c of an effective force generating component. In a motile cell, the active tension depends on the cooperative action of a large number of interacting cytoskeletal components that are regulated by an upstream signaling network. As many of these interactions and their governing rate constants are not known, the present phenomenological model lumps their joint action into a single effective activatory component c. It represents the concentration of typical cell front markers, such as active Ras, PIP_3, or freshly polymerized actin, that are associated with regions of protrusive activity at the cell front. For the dynamics of c we previously proposed a noisy bistable reaction-diffusion system that is confined inside the two-dimensional domain of the cell, and thus is coupled to the phase field in the following way (Alonso et al., 2018; Moreno et al., 2020),

$$\frac{\partial(\phi c)}{\partial t} = \phi[k_a \, c\,(1-c)(c-\delta(c)) - \rho\, c] + F_R\, \phi\, \xi(x,t) + \nabla\left(\phi D \nabla c\right) . \tag{A3}$$

A bistable system seems a natural choice to capture observations, where, due to the presence of waves, the bottom cortex of the cell shows coexisting regions of low and high F-actin concentration. For the associated nonlinearity in the kinetics of c several candidate processes have been identified, such as a positive feedback loop involving Ras, PI3K and F-actin (Sasaki et al., 2007), or the autocatalytic enhancement of actin polymerization due to branching. At the whole cell level, the reaction rate k_a controls the cell's speed, polarity, and persistence of motion, as has been systematically studied earlier (Alonso et al., 2018). The parameters ρ and D designate the degradation rate and the diffusion coefficient of c, respectively. For the

noise an Ornstein-Uhlenbeck dynamics was chosen,

$$\frac{d\xi}{dt} = -k_\eta \, \xi + \eta \, , \tag{A4}$$

which means that temporal correlations in the fluctuations of c decay exponentially with k_η^{-1}. Here, η is a Gaussian white noise with zero mean $\langle \eta \rangle = 0$ and a variance of $\langle \eta(\mathbf{x}, t)\eta(\mathbf{x}', t') \rangle = 2\sigma^2 \delta(\mathbf{x} - \mathbf{x}')\delta(t - t')$. In contrast to previous versions of the model (Alonso et al., 2018; Moreno et al., 2020), the noise appears over the entire ventral surface of the cell (Flemming et al., 2020) and is modulated by a prefactor of $F_R = 1.77(1 - Q)^2$, where Q denotes the fraction of the cell area covered with the concentration c,

$$Q = \frac{\int \phi \, c \, dA}{\int \phi \, dA} \, . \tag{A5}$$

Here, $Q = 1$ corresponds to a cell completely covered with a high level of c and $Q = 0$ indicates vanishing concentrations of c across the cell area. The prefactor F_R ensures that the noise is reduced with decreasing concentration c, and the value $F_R = 1$ is recovered for $Q = 0.25$ as previously employed (Moreno et al., 2020).

In the previous version of our model, the fraction of the cell area that is covered by wave patches of high values of c was regulated to match a fixed value of Q_0 on average. This was achieved by dynamically tuning the quantity $\delta(c)$ in Eq. (A3), which controls whether wave patches grow or shrink (Alonso et al., 2018). Depending on the choice of Q_0, amoeboid (small Q_0) or fan-shaped cells (large Q_0) were observed in numerical simulations of the model (Moreno et al., 2020). However, the experimental observations reported in this article suggest that cells can spontaneously switch between both modes of locomotion. We therefore extended the model such that Q_0 is no longer a fixed parameter but may switch in a noise induced fashion between small and large values, depending on the current relative coverage of the cell area with c as given by the quantity Q,

$$Q_0 = \begin{cases} 0.25, & \text{if } Q \leq Q_{Th} \\ 0.75, & \text{if } Q \geq Q_{Th} \, , \end{cases} \tag{A6}$$

where the parameter Q_{Th} may be chosen between 0 and 1. For large Q_{Th} patches of high c remain small, cells move in an amoeboid fashion, and switches to the fan-shaped state are rare. In contrast, for small values of Q_{Th} most of the cell area will be covered by high values of c driving the cell into fan-shaped motion. To elucidate the role of the newly introduced threshold parameter Q_{Th} for the switching process, we have systematically scanned Q_{Th} between 0 and 1 in steps of 0.1, see Fig. 6.

In addition, the quantity $\delta(c)$ that determines whether patches of large c are growing or shrinking is now dynamically adapting to changes in Q_0 with a characteristic time scale τ_δ according to the following equation,

$$\tau_\delta \frac{\partial \delta(c)}{\partial t} = -\delta(c) + \delta_0 + M \left(\int \phi \, c \, dA - Q_0 \int \phi \, dA \right) \, . \tag{A7}$$

Here, $\delta_0 = 0.5$ corresponds to the neutral situation, where patches of c are neither growing nor shrinking, and the parameter M determines how tightly the coverage with c is regulated to the target value of Q_0.

For the simulations presented here, we used the previously established set of model parameters without any further adaptation to the present situation (Moreno et al., 2020). Only those parameters that are related to the extension of the model were newly chosen. Specifically, the time scales of cell area and wave

area variations, τ_β and τ_δ, were set to $0.2\ s^{-1}$ to match the dynamics observed in our experiments. The concentration constraint parameter M and the threshold value Q_{Th} were systematically changed to explore their impact on the switching behavior, see Fig. 6 and the corresponding part of the Discussion. For the area constraint parameter β_C we did not perform a systematic scan because the dynamics in the cell area fluctuations is not critical for the switching between amoeboid and fan-shaped cells. All model parameters are displayed in Table S1 in the Supplementary Material.

REFERENCES

Alonso, S., Stange, M., and Beta, C. (2018). Modeling random crawling, membrane deformation and intracellular polarity of motile amoeboid cells. PLOS One 13, e0201977. doi:10.1371/journal.pone.0201977

Aranson, I. S. (ed.) (2016). Physical Models of Cell Motility. Biological and Medical Physics, Biomedical Engineering (Cham: Springer International Publishing). doi:10.1007/978-3-319-24448-8

Cao, Y., Ghabache, E., Miao, Y., Niman, C., Hakozaki, H., Reck-Peterson, S. L., et al. (2019). A minimal computational model for three-dimensional cell migration. Journal of The Royal Society Interface 16, 20190619. doi:10.1098/rsif.2019.0619

Devreotes, P. N., Bhattacharya, S., Edwards, M., Iglesias, P. A., Lampert, T., and Miao, Y. (2017). Excitable Signal Transduction Networks in Directed Cell Migration. Annual Review of Cell and Developmental Biology 33, 103–125. doi:10.1146/annurev-cellbio-100616-060739

Flemming, S., Font, F., Alonso, S., and Beta, C. (2020). How cortical waves drive fission of motile cells. Proceedings of the National Academy of Sciences 117, 6330–6338. doi:10.1073/pnas.1912428117

Honda, G., Saito, N., Fujimori, T., Hashimura, H., Nakamura, M. J., Nakajima, A., et al. (2021). Microtopographical guidance of macropinocytic signaling patches. Proceedings of the National Academy of Sciences 118, e2110281118. doi:10.1073/pnas.2110281118

Imoto, D., Saito, N., Nakajima, A., Honda, G., Ishida, M., Sugita, T., et al. (2021). Comparative mapping of crawling-cell morphodynamics in deep learning-based feature space. PLOS Computational Biology 17, e1009237. doi:10.1371/journal.pcbi.1009237

Kulawiak, D. A., Camley, B. A., and Rappel, W.-J. (2016). Modeling contact inhibition of locomotion of colliding cells migrating on micropatterned substrates. PLOS Computational Biology 12, e1005239. doi:10.1371/journal.pcbi.1005239

Löber, J., Ziebert, F., and Aranson, I. S. (2014). Modeling crawling cell movement on soft engineered substrates. Soft Matter 10, 1365–1373. doi:10.1039/c3sm51597d

Löber, J., Ziebert, F., and Aranson, I. S. (2015). Collisions of deformable cells lead to collective migration. Scientific Reports 5, 9172. doi:10.1038/srep09172

Moreno, E., Flemming, S., Font, F., Holschneider, M., Beta, C., and Alonso, S. (2020). Modeling cell crawling strategies with a bistable model: From amoeboid to fan-shaped cell motion. Physica D: Nonlinear Phenomena 412, 132591. doi:10.1016/j.physd.2020.132591

Moure, A. and Gomez, H. (2019). Phase-field modeling of individual and collective cell migration. Archives of Computational Methods in Engineering 28, 311–344. doi:10.1007/s11831-019-09377-1

Najem, S. and Grant, M. (2013). Phase-field approach to chemotactic driving of neutrophil morphodynamics. Physical Review E 88, 034702. doi:10.1103/physreve.88.034702

Sasaki, A. T., Janetopoulos, C., Lee, S., Charest, P. G., Takeda, K., Sundheimer, L. W., et al. (2007). G protein–independent Ras/PI3K/F-actin circuit regulates basic cell motility. The Journal of Cell Biology 178, 185–191. doi:10.1083/jcb.200611138

Shao, D., Levine, H., and Rappel, W.-J. (2012). Coupling actin flow, adhesion, and morphology in a computational cell motility model. Proceedings of the National Academy of Sciences 109, 6851–6856. doi:10.1073/pnas.1203252109

Shao, D., Rappel, W.-J., and Levine, H. (2010). Computational Model for Cell Morphodynamics. Physical Review Letters 105, 108104. doi:10.1103/PhysRevLett.105.108104

Winkler, B., Aranson, I. S., and Ziebert, F. (2019). Confinement and substrate topography control cell migration in a 3D computational model. Communications Physics 2, 1–11. doi:10.1038/s42005-019-0185-x
